# Functional Specificity of Astrocyte Subtypes in Alzheimer’s Disease: Decoding Disease Mechanisms Through Network-based Analysis of Integrated Single-Nuclei Multi-Omic Data

**DOI:** 10.1007/s12035-025-04965-8

**Published:** 2025-04-29

**Authors:** Atılay İlgün, Tunahan Çakır

**Affiliations:** Department of Bioengineering, Gebze Technical University, Kocaeli, Turkey

**Keywords:** Alzheimer’s disease, Astrocytes, Single-nuclei sequencing, Multi-omics, Transcriptome, Protein–protein interactions

## Abstract

Alzheimer’s disease (AD) is the most common cause of dementia. Recent studies have revealed incontrovertible roles of astrocytes in the pathology of AD. Considering the conflicting behaviours of astrocytes in AD brain, they have been proposed to have subtypes. In this study, astrocytes from two publicly available single-nuclei transcriptome datasets were integrated to provide in-depth characterization of astrocyte subtypes in AD. Differentially expressed genes within each astrocyte subtype were analyzed by mapping them onto a human protein–protein interaction network to discover subnetworks with biologically relevant genes. Integrating single-nuclei datasets and using network-based analysis approach led to higher sensitivity in capturing AD-related genes compared to traditional approaches. One of the identified subtypes was highly representative of neurotoxic reactive astrocytes in AD. The results show that A1 reactive astrocytes could have an enhancing role for the amyloid beta and neurofibrillary tangle accumulation through MAPK10, MAPT, and TMED10, which were all found to be differentially expressed in this subtype during AD in our analysis. Moreover, single-nuclei ATAC-Seq data from the same tissue was re-analyzed to evaluate astrocyte subtypes at multi-omic level. It was found that astrocyte subtypes underwent epigenetic reprogramming during AD. Potential transcription factors were also identified for the regulation of the genes that exhibited alterations in both promoter accessibility and gene expression in AD. Comparative analysis of single-nuclei RNA-Seq and ATAC-Seq datasets showed that PTN gene, which was reported to be important for AD pathology, is likely regulated by ATF3 transcription factor in subtype-specific manner in astrocytes.

**Supplementary Information:**

The online version contains supplementary material available at 10.1007/s12035-025-04965-8.

## Introduction

Alzheimer’s disease (AD) is a multifactorial neurodegenerative disorder that affects millions of lives directly and indirectly and knocks back economies of countries worldwide revealing the devastating socio-economic sides of the disease. It is characterized by abnormal accumulation of amyloid-beta (Aβ) and hyperphosphorylated tau proteins as well as synaptic loss [1] and neuronal degeneration, leading to cognitive impairment. Although AD is considered to be a neuron-based disorder, the fact that risk factor genes are also mainly expressed in glial cells reveals their undeniable roles on AD pathology. Astrocytes perform variety of functions in brain, regulating ion homeostasis, neurotransmitter clearance, and preventing excitotoxicity in the central nervous system (CNS) [2]. They also regulate synapse formation, synaptic transmission, and synapse maintenance [2], with the ability to eliminate synapses by phagocytosis [3]. They are involved in the structure of blood–brain barrier (BBB) by their end-feet, together with endothelial cells and pericytes. Astrocytes also provide trophic factors and energy to neurons.

Abnormal accumulation of Aβ in intercellular space is one of the hallmarks of AD and has been shown to alter many different biological processes in astrocytes including neurotransmitter clearance, calcium signalling, and gliotransmission [4]. These altered biological processes might worsen the disease pathology, but astrocytes also have a variety of mechanisms to clear and eliminate Aβ. For example, they express AQP4 (Aquaporin 4) water channels in their end-feet, and, by this way, they are involved in amyloid beta clearance [5]. Moreover, Aβ clearance is also mediated by APOE, CLU, ACT, and α2-macroglobulin proteins in astrocytes [6]. Astrocytes also express proteases (ECE1, ECE2, NEP, IDE) that can degrade Aβ peptide into smaller fragments [7]. They were found to be closely associated with amyloid plaques in human brain entorhinal cortex, and this might be the sign of their neuroprotective roles [6]. Despite the fact that astrocytes modulate events related to Aβ, thereby acting as neuroprotective, in theory, due to the astrocytic upregulation of amyloid precursor protein (APP) and β-secretase- 1 during AD, they can also contribute to Aβ production [8]. Moreover, neuroinflammation can promote the production of astrocytic amyloid beta [8]. Also, it was shown that Aβ is able to activate NF-κB signalling pathway in astrocytes, which eventually changes phagocytic properties of microglia and disrupts neuronal morphology through the upregulation of C3 protein [9]. As a summary, astrocytes can have both neuroprotective and neurotoxic roles in AD.

A wide variety of astrocytic functions in CNS should be evaluated in more detail to reveal the neuroprotective or neurotoxic aspects of astrocytes. A possible explanation for their heterogenous roles in AD is that astrocytes might indeed have subtypes. For proper characterization of astrocyte subtypes, comprehensive single-cell genomics approaches and high amount of astrocytes were required because small populations cannot adequately reflect the transcriptional dynamics during AD. Although astrocytes are one of the most abundant cell types in CNS, low capturing rate of astrocytes causes insufficient reflection of the disease conditions. For this reason, Sadick et al. (2022) developed an astrocyte enrichment strategy to increase the number of captured astrocytes up to tenfold per donor and conducted single-nuclei transcriptomic profiling of post-mortem human brain prefrontal cortex astrocytes [10]. They profiled more than 40,000 astrocytic nuclei from healthy and AD conditions and identified nine astrocyte subtypes with unique transcriptional signatures. On the other hand, by profiling around 10,000 astrocyte nuclei for chromatin sequencing from the same tissue, Morabito et al. provided an excellent source for the identification of the epigenetic alterations in astrocytes during AD [11].

In this study, publicly available single-nuclei transcriptome datasets [10, 11] based on post-mortem human brain prefrontal cortex from AD patients and healthy controls were re-analyzed, and astrocytes from both datasets were integrated to provide in-depth characterization of astrocyte subtypes in AD. Then, differentially expressed genes (DEGs) between AD and control astrocytes within each subtype were mapped onto a human protein–protein interaction network to generate subnetworks with biologically relevant genes. The findings were reinforced by analyzing snATAC-seq-based astrocytes data available for the same tissue in one of the studies [11], enabling the identification of chromatin accessibility information for the astrocyte subtypes as well as differentially accessible regions.

## Methods

### snRNA-seq and snATAC-seq Data Analysis Overview

**Fig. 1:**
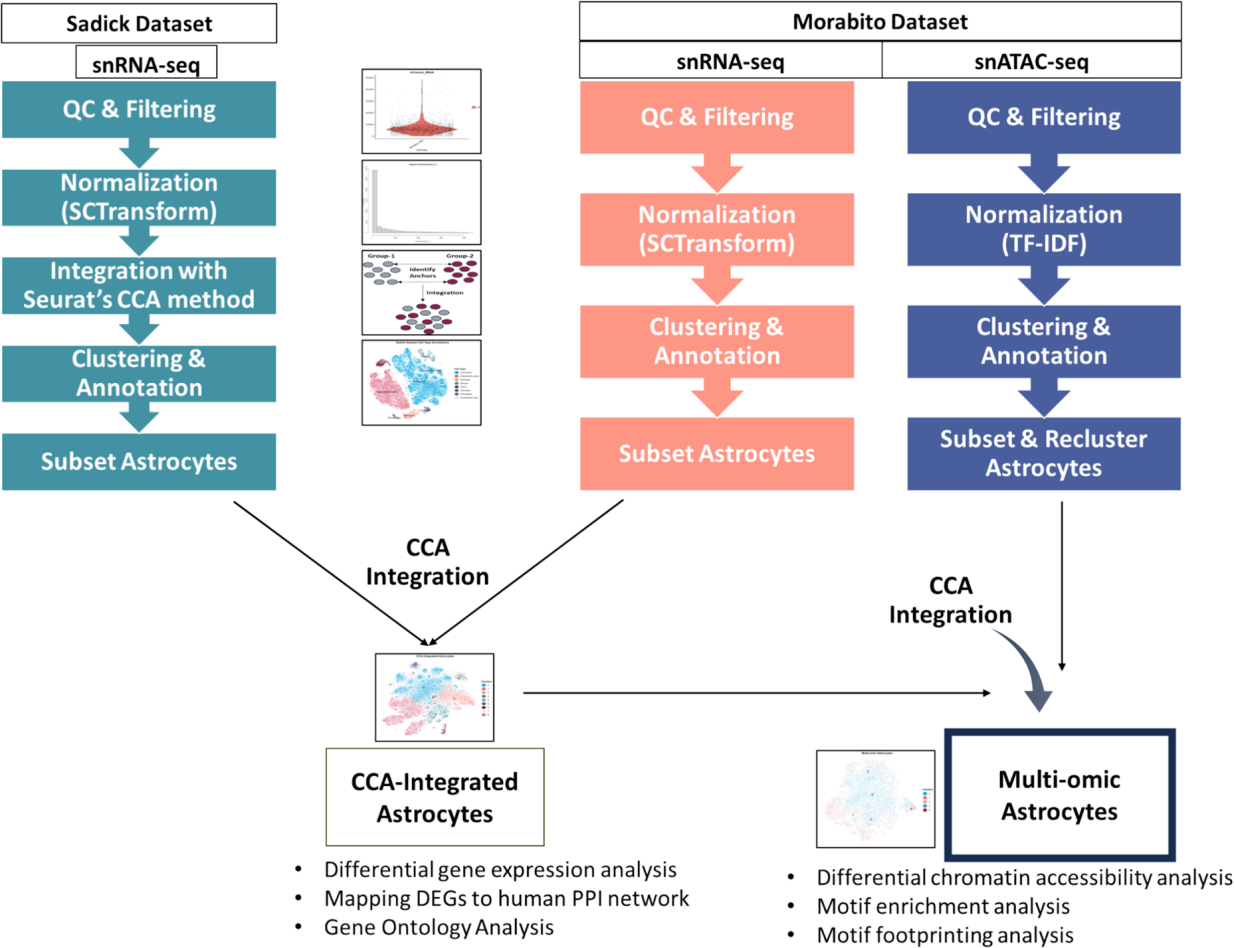
Overview of single-nuclei data analysis approach and overall methodology implemented in this study. Each dataset was analyzed independently, and after the annotation of CNS cells, astrocytes were integrated using CCA. The integrated data was used to identify DEGs between control and AD groups, followed by their mapping onto the human PPI network. In parallel, snATAC-seq dataset was also analyzed, and astrocytes were subsetted and reclustered. Subsequently, multi-modal integration was implemented, and differentially accessible regions were identified. This was followed by motif enrichment and foot printing analyses. QC, quality control; CCA, canonical correlation analysis

In this study, two independent snRNA-seq datasets (GSE167494 and GSE174367) [10, 11] collected from the post-mortem human brain prefrontal cortex tissue of patients with AD and healthy controls were downloaded from the Gene Expression Omnibus (GEO) database. We will refer to these datasets by the names of the first authors in their respective data articles: Sadick and Morabito. While both contain snRNA-seq data, the Morabito study also has snATAC-seq data available for the same tissue. Each dataset consists of both AD and control samples. In the Sadick dataset [10], there are five control and nine AD donors, while the Morabito dataset [11] includes seven control and 11 AD donors. Count and peak matrices of the datasets were imported to R (v4.2.1). The single-nuclei RNA-seq datasets were processed by the comprehensive single-cell genomics tool Seurat (v4.3.0) [12]. The single-nuclei ATAC-seq dataset was processed by using Signac (v1.7.0) [13], the single-cell chromatin data analysis tool. The two single-nuclei RNA-seq datasets were analyzed independently. For the normalization of the snRNA-seq datasets, sctransform::SCTransform (v0.3.5) [14] function was used. After the annotation of the major cell types in each dataset, astrocytes were subsetted, integrated, and reclustered to determine the subtypes. Meanwhile, snATAC-seq data was processed, annotated, subsetted, and reclustered. Then, Seurat’s label transfer approach, called canonical correlation analysis (CCA) [15], was used to transfer subtype labels in RNA-seq astrocytes to ATAC-seq astrocytes. This enabled chromatin accessibility information for the astrocyte subtypes. Since all the three datasets (2 snRNA-seq and 1 snATAC-seq) were processed independently, analysis steps of each of them are explained independently in the following sections in detail. Figure 1 gives the graphical representation of the whole methodology. Online Resource 1 provides further information about the quality control, clustering, and integration parameters.

### Integration of Astrocytes Obtained from the Sadick and Morabito Datasets by CCA

Firstly, the subsetted astrocytes from the Sadick and Morabito datasets were imported into R. All assays and slots, except the RNA assay that stores raw counts, were removed from the Seurat objects. Then, the count matrices of astrocytes from each donor were independently normalized with sctransform::SCTransform function. For each donor, 2000 variable features were selected, and percentages of mitochondrial, ribosomal, and heat-shock proteins as well as hemoglobin transcripts were regressed out during this normalization step. Then, by using Seurat::SelectIntegrationFeatures function, features that will be used in the integration of donors were selected and mitochondrial, ribosomal, and heat-shock proteins as well as hemoglobin transcripts were removed among these variable features (1991 variable genes remained). Subsequently, the normalized data was prepared for CCA by Seurat::PrepSCTIntegration function. Integration by CCA was performed using Seurat::FindIntegrationAnchors and Seurat::IntegrateData functions, respectively (dims = 50 and k.weight = 60). After the integration, linear (PCA) and non-linear dimension reductions (tSNE) were performed by using the first 50 PCs. Then, Shared Nearest-Neighbors (SNNs) were computed, and the Louvain algorithm was used for clustering (0.15 resolution), leading to the formation of nine subclusters. After the clustering, Wilcoxon rank sum test was used to identify subtype-enriched transcripts.

### Differential Gene Expression Analysis for Astrocyte Subtypes

After identifying the subtypes of astrocytes, DEGs were determined by comparing AD and control astrocytes in each subtype independently. For the DEG analysis, DESeq2 [16] and ZINB-WaVE [17] R packages were used in combination as our integrated snRNA-seq data was zero-inflated (see below). The DEG analysis was performed for each subtype as well as ignoring the subtype information (see Online Resource 4 for differential testing results). For cluster-specific differential testing, cluster-specific Seurat objects were generated, and differential testing processes were conducted using cluster-specific Seurat objects, thereby, steps given below were implemented into each Seurat object. Firstly, the Seurat object of the CCA-integrated astrocytes was converted into SingleCellExperiment (SCE) object. Genes were kept if they had a total count of at least six cells, and other genes were removed. After filtering out the lowly expressed genes, zinbwave::zinbwave function was run to extract low-dimensional signal from zero-inflated data, with the following parameters: K = 0, epsilon = 10^12^, observationalWeights = TRUE. Subsequently, size factors were estimated by using scran::computeSumFactors function in the scran R package (v1.24.1) [18]. Lastly, DESeq2::DESeq function was run with the following parameters optimized for snRNA-seq data [19–21]: test = LRT, minmu = 10^−6^, minRep = Inf, reduced = 1. Genes were considered as differentially expressed if they had Benjamini–Hochberg adjusted *p*-value ≤ 0.05 and considered as upregulated and downregulated if FC > 1.20 and FC < 0.83, respectively.

### KeyPathwayMiner and Functional Enrichment Analysis

After the identification of DEGs in each subtype between control and AD cells, these genes were used as input to the KeyPathwayMiner (KPM) algorithm [22], which is a method to discover subnetworks in a given biological interaction network. By this network-based approach, DEGs were mapped onto a human protein–protein interaction (PPI) network. The human PPI network (version 4.4.222) was downloaded from the BioGRID database. Only the physical interactions and human–human-based interactions were kept in the network. Prediction-based interactions were also removed from the network, leading to 18,926 proteins and 1,065,083 interactions. KPM was run for each subtype (see Online Resource 4 for network-based analysis results). For this study, KPM Standalone v5.1 was used with the following parameters: L1 = 0, strategy = INES, algo = GREEDY, maxSolutions = 1, K = 5. Subsequently, the genes in the subnetworks discovered by KPM were subjected to functional enrichment analysis using gprofiler2 [23] to determine subtype-specific molecular patterns and responses to AD. Gprofiler2::gost function in R was used to run the enrichment analysis. Cytoscape [24] was used for the visualization of interactions.

### snATAC-seq Data Processing and Reclustering of ATAC-seq Astrocytes

The peak matrix of the Morabito snATAC-seq dataset [11] was introduced into the R environment. Total number of peaks, nucleosomal signal, and TSS (transcriptional start site) signal are commonly used quality control metrics for snATAC-seq data. These metrics were assessed, and cells in donors were filtered based on these metrics as Seurat recommended (see Online Resource 1 J-O for the distribution of unfiltered and filtered quality control metrics for the Morabito snATAC-seq dataset). For normalization and dimension reduction, the LSI (latent semantic indexing) method was used. Signac::RunTFIDF, Signac::FindTopFeatures, and Signac::RunSVD functions were run for normalization, selection of highly variable peaks, and dimension reduction respectively. Signac::DepthCor and Seurat::ElbowPlot functions were used to determine the number of LSI components to be used in downstream analyses. The first LSI dimension keeps technical variation due to sequencing depth most of the time; therefore, it was removed. Then, the non-linear dimension reduction method tSNE was implemented (dims = 2:15) and followed by Seurat::FindNeighbors and Seurat::FindClusters functions (0.1 resolution and the Louvain algorithm). Subsequently, the logistic regression (LR) framework was implemented by using Seurat::FindAllMarkers function to determine the differentially accessible peaks in each cluster (see Online Resource 3 for differential testing results). Criteria for significancy of peaks were the same as above. Identified clusters were annotated (Online Resource 2 F-G) using the differential accessibility of peaks. After the annotation of major CNS cell types, astrocytes were subsetted and reclustered. For the reclustering of the ATAC-seq astrocytes, Signac::RunTFIDF, Signac::FindTopFeatures, and Signac::RunSVD functions were run in order. Then, Signac::DepthCor and Seurat::ElbowPlot functions were used to determine the number of LSI components to be used in the downstream analyses. Then, the non-linear dimension reduction method tSNE was implemented (dims = 2:10), and the Seurat object for the ATAC-seq astrocytes was saved for multi-modal integration.

### snRNA-seq and snATAC-seq Integration

Seurat objects of both CCA-integrated RNA-seq astrocytes (42,020 cells) and pre-processed ATAC-seq astrocytes (9554 cells) were introduced into the R environment. Default assay of CCA-integrated astrocytes was set as RNA assay, and Seurat’s fundamental pre-processing workflow (NormalizeData, FindVariableFeatures, ScaleData, respectively) was implemented into this assay. After identifying 2000 variable features, which will be used for label transfer, mitochondrial, ribosomal, and heat-shock proteins as well as hemoglobin transcripts were removed among those variable features, and 1977 variable features remained. Then, gene activities for these variable features were calculated by using Signac::GeneActivity function, and the calculated gene activities were added as an assay into the Seurat object of ATAC-seq astrocytes and they were log-normalized and scaled by Seurat::NormalizeData and Seurat::ScaleData, respectively. Then, CCA was performed by using Seurat::FindTransferAnchors function (reduction = cca) to identify anchors between datasets. Afterwards, Seurat::TransferData function was used to transfer the cell type labels (dims = 2:30). Five of the subtype labels in the RNA-seq astrocytes (clusters 0, 1, 2, 3, and 6) were successfully transferred into the ATAC-seq astrocytes (5632, 2618, 954, 203, and 144 cells identified for the given clusters, respectively) (Online Resource 2I-J). In order to check the quality of the label transfer process, RNA-seq and ATAC-seq astrocytes were co-embedded (Online Resource 2 K). For the co-embedding, the gene expression matrix of RNA-seq astrocytes (in RNA assay) was imputed into ATAC-seq astrocytes based on the previously computed anchors. Then, the snRNA-seq and snATAC-seq astrocyte Seurat objects were merged. After the merging, the dataset was centered by using Seurat::ScaleData function. Then, PCA was implemented by Seurat::RunPCA function, and 30 PCs were used in the downstream analyses. After the linear dimension reduction, the Harmony R package (v0.1.1) [25] was used to remove batch effects across snRNA-seq and snATAC-seq datasets. After the batch correction, the non-linear dimension reduction method tSNE was implemented by using the Harmony embeddings (Online Resource 2 K). Low dimensional representation of RNA- and ATAC-seq astrocytes, prediction scores, and correlation between promoter accessibility and gene expression show the accuracy of the label transfer process (Online Resource 2I-K).

### Differential Chromatin Accessibility, Motif, and Motif Footprinting Analysis

For differential chromatin accessibility analysis, DESeq2 was used with the same parameters used for the snRNA-seq data due to the similarities in the distributions of the snRNA-seq and snATAC-seq datasets. We only used peaks that fell into the promoter regions for the differential testing. We identified 14,117 unique peaks in the promoter regions, and all chromatin accessibility analyses were conducted using these peaks. After the differential testing, Signac::ClosestFeature function was used to annotate the peaks. The results were considered to be differentially accessible if *p*-value < 0.01 and FC > 1.20 or < 0.83.

For a set of selected genes that exhibited both differential expression and differential chromatin accessibility, we conducted motif analysis to determine their potential regulators, e.g., transcription factors (TFs). We focused on TFs that were found to be differentially expressed during AD in our snRNA-seq analysis. We identified differentially expressed transcription factors in each cluster (clusters 0, 1, 2, 3, and 6), and we implemented cluster-specific motif analysis for these transcription factors in their respective clusters. Firstly, we downloaded motif position and frequency matrices from the JASPAR2022 database, and this information was added into our Seurat object. For each subtype identified in the snATAC-seq data, we identified accessible regions by using Signac::Accessible Peaks function and we selected peaks that fell into the promoter regions. Then, we used Signac::GetMotifData function to obtain potential promoter regions that our TF of interest can bind to. Moreover, Signac::Footprint function was used for TF footprinting analysis, and by this way, we validated the binding of specific TFs in their respective clusters.

## Results

### Integration of Astrocytes from Two Different Datasets Reveals Transcriptomic Differences Between Subtypes

**Fig. 2:**
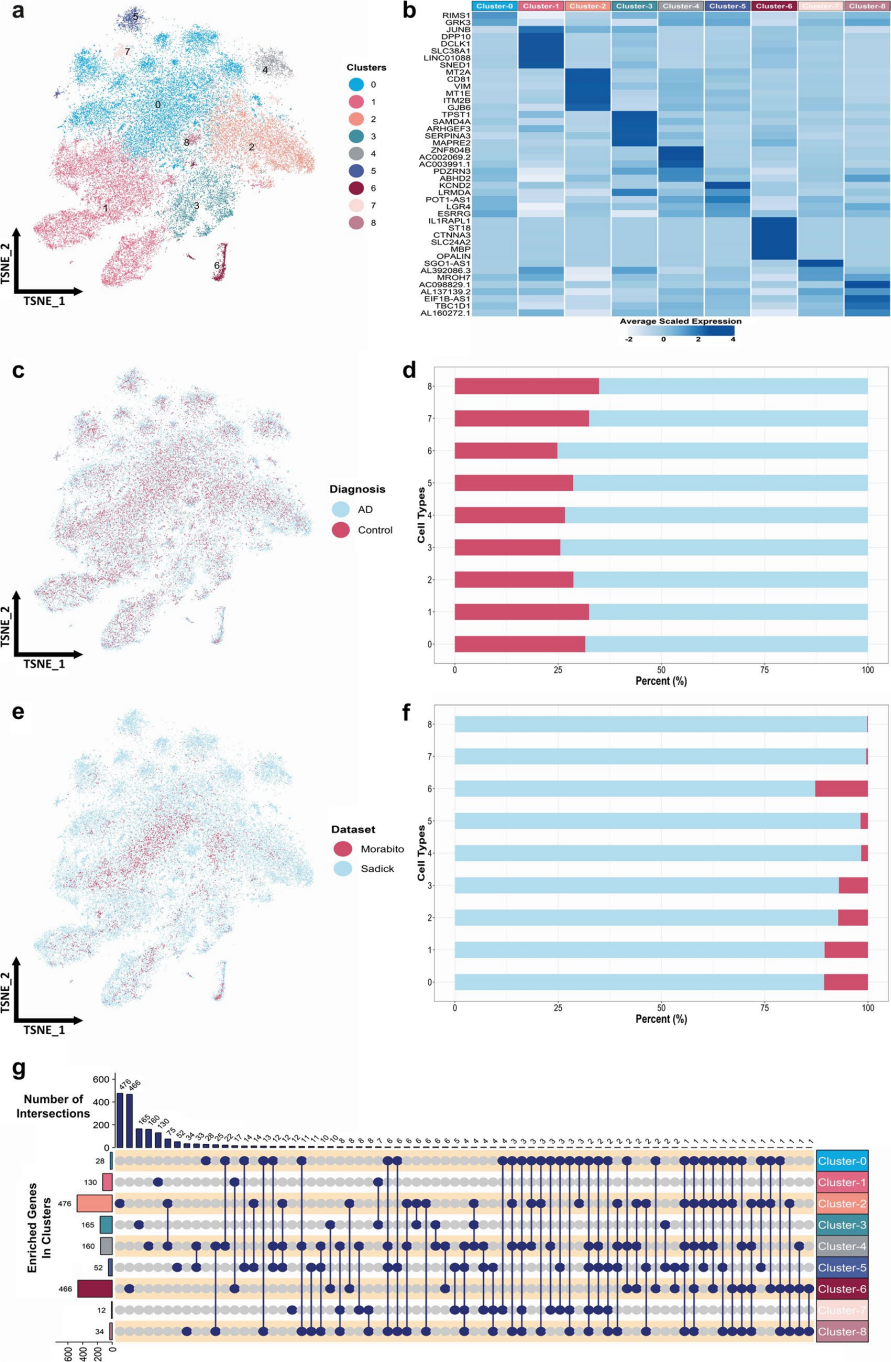
Integration of astrocytes from two different datasets reveals transcriptomic differences between subtypes. **a** tSNE plot of astrocyte subtypes (42,020 nuclei). **b** Average scaled expression of uniquely enriched genes in astrocyte subtypes. **c**, **d** Diagnosis- and **e**, **f** dataset-based distribution of astrocyte subtypes. **g** Upset diagram of intersection of enriched genes in astrocyte subtypes

A total of 38,207 and 3813 cells were obtained from the Sadick and Morabito datasets, respectively, resulting in 42,020 integrated astrocytes (Fig. 2a) and nine transcriptomically distinct subtypes (Fig. 2a and b). Each astrocyte subtype was assigned a colour for proper visualization. These subtypes were verified to be not driven by any batch such as dataset, disease state (Fig. 2c–f), donor, sex, and age (Online Resource 2 F). tSNE and bar plots show dataset- and disease-based distributions of cells, validating the proper integration of astrocytes (Fig. 2c–f). In all subtypes, the percentages of AD astrocytes were over 60%. Since the number of astrocytes obtained from the Morabito dataset was low compared to the Sadick dataset, some subtypes are predominantly represented by the Sadick dataset (Fig. 2f).

Among 42,020 cells, 29,153 were AD astrocytes and 12,867 were healthy astrocytes (Online Resource 2 F). Non-parametric Wilcoxon rank sum test was conducted to observe the transcriptional differences between the astrocyte subtypes, and genes significantly highly expressed in each subtype compared to all other subtypes were identified. Such genes were termed enriched genes. The number of uniquely enriched genes in each cluster and the intersection of enriched genes between clusters were given in Online Resource 2 F and Fig. 2g, respectively. The highest number of enriched genes were identified in clusters 2 and 6, with 476 and 466 genes, respectively. While 425 of 466 genes were uniquely enriched in cluster 6, 391 of 476 genes were uniquely enriched in cluster 2. A heatmap visualizing the top five uniquely enriched genes in each subtype based on the fold-change value was given in Fig. 2b. GRK3 (G protein-coupled receptor kinase 3), which was shown to be expressed by astrocytes in human AD hippocampus [26], was uniquely enriched in cluster- 0 astrocytes. Glutamine transporter SLC38A1 (SNAT1) was previously reported to be downregulated during AD [27], and it was uniquely enriched in cluster- 1 astrocytes. Glutamate release from presynaptic terminals was shown to be decreased in human AD brains, leading to impaired synaptic transmission among glutamatergic neurons [27]. As glutamate-glutamine cycle is crucial for proper synaptic transmission, enriched SLC38A1 expression in cluster 1 might be important for protective pathology. VIM (Vimentin) and SYNM (Synemin) are intermediate filament proteins and contribute to astrocyte cytoskeleton, and they were uniquely enriched in clusters 2 and 3, respectively. SERPINA3 (Serpin Family A Member 3) was found to be upregulated in human frontal cortex samples in early AD, and it was shown to be predominantly produced by reactive astrocytes, which surround the senile plaques [28, 29]. SERPINA3 was found to be uniquely enriched in cluster- 3 astrocytes and might be contributing to AD pathology. APOE (apolipoprotein E), the major risk factor gene for idiopathic late-onset AD, was enriched in clusters 2 and 4. Similarly, APP (amyloid precursor protein) is uniquely enriched in cluster 6. Apart from the uniquely enriched transcripts, there are also similarities across subtypes. The highest number of commonly enriched genes was detected between clusters 2 and 4 (75 genes), followed by 33 genes between clusters 4 and 5. Moreover, 12 genes were commonly detected between clusters 0, 4, and 5, and 11 genes were commonly detected between clusters 0, 4, and 8 (Fig. 2g).

### Single-Nuclei Astrocyte Integration Followed by Human PPI Network Mapping Captures AD-Related Genes More Sensitively Compared to Traditional Approaches

Differential gene expression analyses conducted to compare AD and control astrocytes in each cluster revealed upregulated and downregulated genes during AD. These genes were mapped on the human protein–protein interaction network using KeyPathwayMiner [22], and subnetworks with differentially up/downregulated genes were discovered. Our aim was to map DEGs onto the human protein–protein interaction network to create a subnetwork with more biologically relevant genes since this approach filters the list of DEGs such that those forming a physically interacting subnetwork are reported. The numbers of up and downregulated elements (DEGs and subnetwork genes) for each subtype during AD were given in Table 1. Additionally, a comprehensive AD-related gene list was compiled by our group previously [30], which was composed of 772 genes (see Online Resource 4), by using the combination of a set of genes obtained from GeneCards, Gene Ontology, and KEGG databases and a study based on literature curation. This list was used to identify AD-related genes in our clusters (see Table 1). Moreover, all AD and all healthy astrocytes were also compared by ignoring subtypes (referred to as subtype-unaware comparison) to validate the benefit of the subtype-specific approach.

**Table 1:** Number of differential expressed elements in astrocyte subtypes. “DEG” column shows the number of DEGs, and “Subnetwork Genes” column shows the number of DEGs with physically interacting corresponding proteins, obtained after mapping DEGs onto the human PPI network. The number of AD-related genes identified in each gene set is also given (DEGs, differentially expressed genes; AD-Rel., Alzheimer’s disease-related genes)

	DEG			Subnetwork genes		
Clusters	Up	Down	AD-Rel.	Up	Down	AD-Rel.
Cluster 0	458	275	32	299	137	26
Cluster 1	470	342	44	334	181	36
Cluster 2	542	211	55	406	109	48
Cluster 3	449	426	41	310	296	32
Cluster 4	282	162	20	212	91	17
Cluster 5	233	135	19	172	84	15
Cluster 6	322	243	29	244	146	20
Cluster 7	175	127	10	132	78	8
Cluster 8	144	125	9	103	72	7
Subtype-unaware	723	394	51	485	185	45

A total of 107 unique AD-related genes were obtained from the combination of all subtype-specific DEG results, and 94 of them were the part of the subtype-specific subnetworks. On the other hand, the “subtype-unaware” comparison of AD and control astrocytes identified 51 differentially expressed AD-related genes, 45 of which appeared in the discovered subnetwork. The majority of AD-related genes identified in the subtype-specific differential gene expression analysis (69/107 genes) could not be captured by the subtype-unaware approach, and this reveals the importance of the subtype-specific approach. Online Resource 4 gives the list of AD-related genes identified in the subnetwork-based and DEG-based approaches in a subtype-specific or subtype-unaware manner. Besides, our single-nuclei dataset integration followed by mapping DEGs on the human PPI network was proven to capture AD-related genes more sensitively compared to the original study that generated the data [10] where they also conducted differential gene expression analysis for the nine astrocyte subtypes and identified 65 unique AD-related genes.

### Subtype-Specific Altered Biological Processes and Molecular Functions Were Identified by Gene Ontology Enrichment

**Fig. 3:**
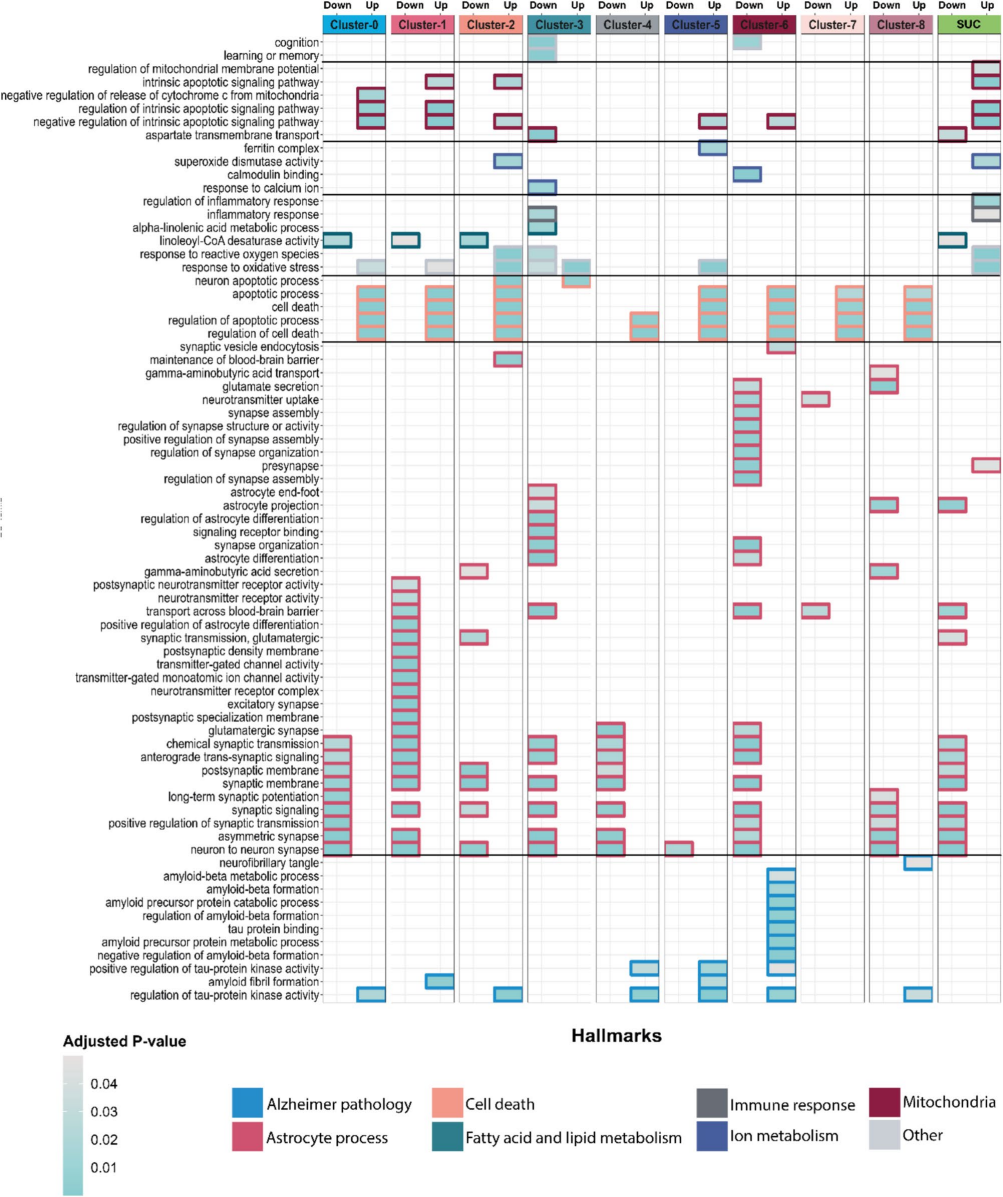
Altered biological processes in astrocyte subtypes during AD. Certain biological processes and functions were found to be altered in astrocyte subtypes during AD. These changes were grouped into several hallmarks. Hallmarks are colour-coded in their frameworks, and colours inside the rectangles change based on the adjusted *p*-value (Benjamini Hochberg corrected) in their respective gene ontology analysis. SUC, subtype-unaware comparison

Subnetwork genes identified in the previous section were subjected to gene ontology analysis for the identification of subtype-specific altered functions in AD astrocytes. By using the up and downregulated subnetwork genes in each cluster, gene ontology analyses were conducted, and altered biological processes and molecular functions were identified. Identified GO terms were classified based on their involvement in the following major relevant molecular processes: AD pathology, astrocyte process, cell death, fatty acid and lipid metabolism, immune response, ion metabolism, and mitochondria (Fig. 3). Several AD-related biological processes captured with the subtype-specific analysis could not be identified with the subtype-unaware analysis. Also, we identified heterogeneity across the subtypes in AD through the GO analysis.

We found that apoptosis-related GO terms were upregulated in the AD astrocytes in all clusters. Apoptotic process, cell death, regulation of apoptotic process, and regulation of cell death were found to be upregulated nearly in all clusters. Interestingly, we did not identify any apoptosis-related GO terms in the overall AD astrocyte phenotype (e.g., subtype-unaware) despite their abundance in the subtype-specific results. Cognitive impairment is one of the devastating results of AD, and we identified the cognition term to be downregulated in the cluster- 3 and cluster- 6 AD astrocytes. Moreover, learning or memory term was uniquely downregulated in cluster- 3 AD astrocytes.

Response to oxidative stress term was found to be upregulated in AD astrocytes in clusters 0, 1, 2, and 5 and the overall AD astrocyte phenotype. However, in cluster- 3 AD astrocytes, the term has both upregulated and downregulated components. Similarly, response to reactive oxygen species term was found to be upregulated in cluster- 2 but downregulated in cluster- 3 AD astrocytes. Oxidative stress might enhance the disease pathology, thereby the dysregulation/downregulation of oxidative stress response in cluster- 3 AD astrocytes might be neurotoxic.

Astrocytes can directly be involved in Aβ and NFT formation [31]. We found that AD pathology-related GO terms such as amyloid beta metabolic process, amyloid beta formation, amyloid precursor protein catabolic process, regulation of amyloid-beta formation, tau protein binding, amyloid precursor protein metabolic process, and negative regulation of amyloid-beta formation terms were uniquely upregulated in cluster- 6 AD astrocytes. Also, the positive regulation of tau-protein kinase activity was found to be upregulated in cluster- 4, 5, and 6 AD astrocytes. In addition, the neurofibrillary tangle term was found to be uniquely upregulated in cluster- 8 AD astrocytes. Cluster 6 has the highest number of AD pathology-related terms.

Aspartate transmembrane transport term was found to be uniquely downregulated in cluster- 3 AD astrocytes, and this is also the case for the overall AD astrocyte phenotype. In addition, inflammatory response term was found to be downregulated in cluster- 3 AD astrocytes but upregulated in the overall AD astrocyte phenotype. This also reveals the contrast between the subtype-unaware and subtype-specific approaches. Moreover, alpha-linolenic acid metabolic process term was found to be uniquely downregulated in cluster- 3 AD astrocytes. A recent study revealed that alpha-linolenic acid contributes to AD pathology by regulating the susceptibility of BBB integrity [32]. There are also other uniquely identified terms such as maintenance of blood–brain barrier, which was found to be uniquely upregulated in cluster- 2 AD astrocytes and can be neuroprotective. Moreover, ferritin complex term was found to be uniquely upregulated in cluster- 5 AD astrocytes. This might be associated with iron storage, hence contributing to AD pathology. While synaptic signalling-related GO terms were downregulated in almost all clusters, synapse structure, assembly, and organization-related GO terms were uniquely downregulated in cluster- 6 AD astrocytes.

### Human A1 Neurotoxic Reactive Astrocytes Were Identified in Cluster 3

**Fig. 4:**
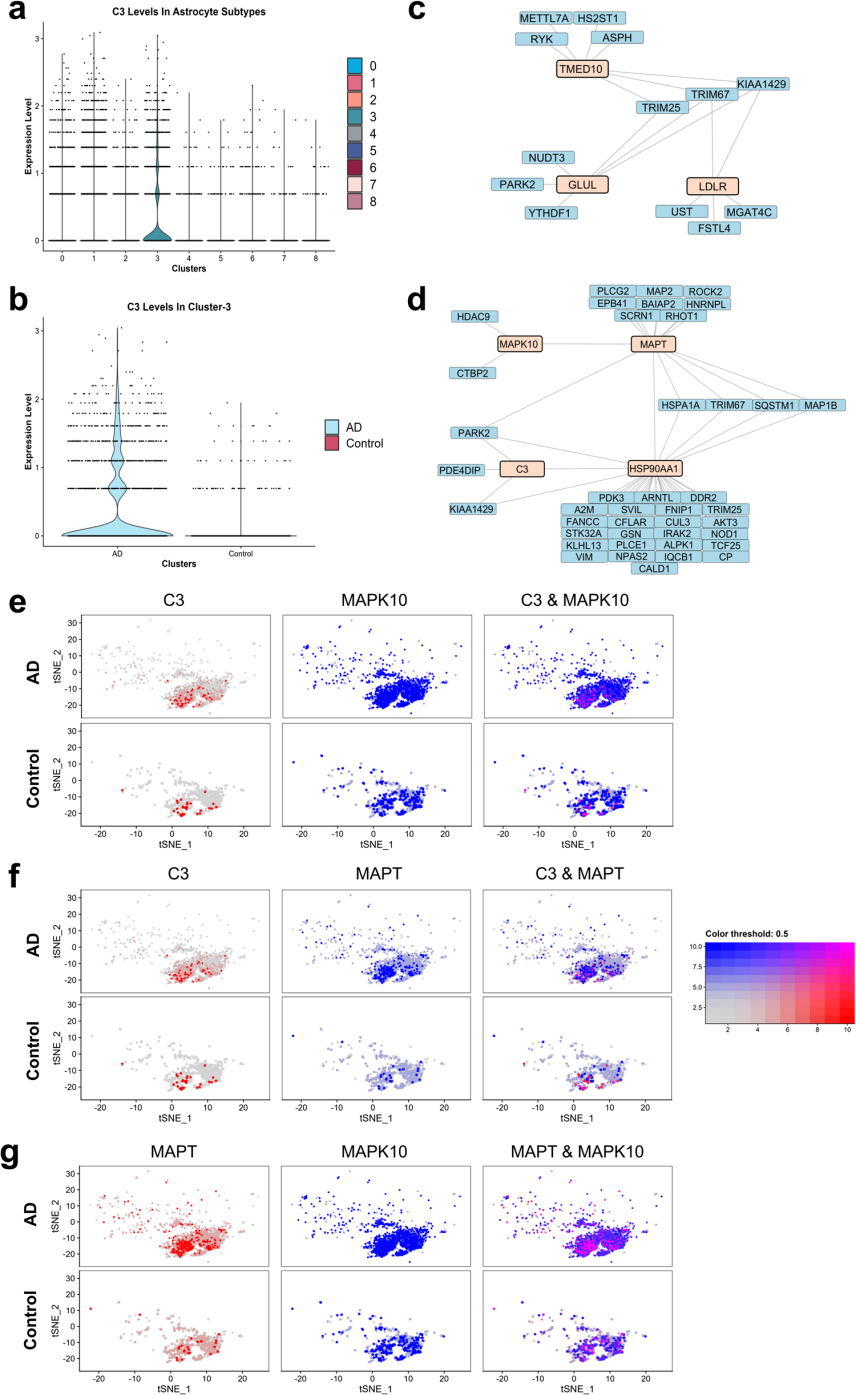
A1 neurotoxic astrocytes were found in cluster 3, and they might increase the NFT burden in AD. **a** C3 expression across all astrocyte clusters. **b** C3 expression in cluster- 3 AD (2515 cells) and control (862 cells) astrocytes. **c** Downregulated PPI subnetwork of cluster- 3. **d** Upregulated PPI subnetwork of cluster- 3. Co-expression plots of **e** C3-MAPK10, **f** C3-MAPT, and **g** MAPT-MAPK10 in cluster- 3 AD and control astrocytes, respectively

In 2017, Liddelow and colleagues [33] reported two types of reactive astrocytes called A1 and A2, and they were generated by neuroinflammation and ischemia, respectively. A1 reactive astrocytes were shown to upregulate many different sets of destructive genes, and therefore, they were considered to be neurotoxic. On the other hand, A2 reactive astrocytes were reported to be neuroprotective as they upregulated neurotrophic factor genes. Complement component 3 (C3) gene was reported to be a marker for A1 reactive astrocytes, and they were detected in post-mortem human brain AD astrocytes [33]. We found that C3 was uniquely enriched in cluster- 3 (Fig. 4a) and found to be significantly upregulated in cluster- 3 AD astrocytes (Fig. 4b). We propose that C3^+^ cells here might be corresponding to A1 neurotoxic reactive astrocytes. We also found that the majority of C3^+^ astrocytes originated from the AD patients (Fig. 4b). Despite the fact that C3^+^ astrocytes were mainly obtained from the AD patients, there were some C3^+^ astrocytes also obtained from the healthy cases. Studies [34, 35] revealed that the increase in the number of C3^+^ astrocytes was also possible with aging, thereby validating our results. In addition to the reported characteristics of A1 reactive astrocytes [33], we also wanted to discover the novel features of them. To this end, we investigated the interactions between the identified uniquely downregulated and upregulated subnetwork genes in cluster 3 by highlighting these genes in PPI networks.

As discussed in detail above, GLUL, LDLR, and TMED10 were found to be uniquely downregulated in cluster 3, and their downregulation was associated with an increase in neurotoxicity during AD. For this reason, we highlighted these genes in the downregulated subnetwork of cluster 3. These genes regulate different functions in astrocytes, and we did not find any direct association between these genes in the network results. Instead, we found an association between these genes through TRIM E3 ubiquitin ligases (Fig. 4c). TRIM25 physically interacts with both TMED10 and GLUL, and TRIM67 interacts with TMED10 and LDLR. TRIM E3 ubiquitin ligases, TRIM25 and TRIM67, were reported to have regulatory roles in brain development, behaviour [36], AD pathology [37], and neuroinflammation [38]. Therefore, they might also be associated with the neurotoxic behaviour of A1 astrocytes.

We also highlighted some of the AD pathology-related genes in the upregulated subnetwork of cluster 3. Since MAPK10 expression was reported to cause an increase in Aβ and NFTs [39] and was found to be uniquely upregulated in cluster- 3 AD astrocytes, we wanted to check the associations of MAPK10 in C3^+^ astrocytes. Although we did not find a direct association between C3 and MAPK10 in the upregulated PPI network of cluster 3, we observed co-expression between C3 – MAPK10 (*R* = 0.16, *p*-value < 2.2 × 10^−16^) (Fig. 4e), and this situation might be indicating the potential role of A1 reactive astrocytes to enhance Aβ and NFT accumulation through MAPK10. Additionally, we found interaction (Fig. 4d) and co-expression (*R* = 0.28, *p*-value < 2.2 × 10^−16^) (Fig. 4g) between MAPK10 and MAPT, which is the tau protein and found to be upregulated in cluster- 3 AD astrocytes. We also found that MAPT was associated with important chaperone HSP90AA1, which is involved in AD pathology, and HSP90AA1 was found to be associated with C3 (Fig. 4d). Furthermore, we did not find a direct association between C3 and MAPT; however, we observed co-expression between C3 – MAPT (*R* = 0.17, *p*-value < 2.2 × 10^−16^) (Fig. 4f). Lastly, TRIM67, which was discussed above, was also found to be interacting with MAPT and HSP90AA1 in the upregulated subnetwork of cluster 3, and the way that it appears in both up and downregulated subnetworks might strengthen its potential to involve in A1 phenotype to modulate AD pathology. Overall, these results indicate that A1 astrocytes might have a strong contribution to Aβ and NFT accumulation.

### Alzheimer’s Disease Leads to Epigenetic Alterations in Astrocyte Subtypes

Changes in the chromatin accessibility can imply altered gene expression. As we added the chromatin accessibility component to the gene expression information, we were able to evaluate the astrocyte subtypes at the multi-omic level and check whether they undergo epigenetic alteration or not. To this end, subtype-specific differentially accessible promoter regions were identified during AD. Then, by using the previously mentioned AD-related gene list compiled from the literature, AD-related promoter regions were determined. The number of differentially accessible promoter regions, AD-related promoter regions, and their corresponding genes are given in Table 2.

**Table 2:** Alzheimer’s disease leads to epigenetic alterations in astrocytes. The terms “increased” and “decreased” correspond to increased and decreased promoter accessibility

Clusters	Increased	Decreased	AD-related increased	AD-related decreased
Cluster 0	78	48	2 (PSMA5, PAICS)	1 (SLC19A1)
Cluster 1	79	60	6 (NTRK1, TP63, IL12B, OLR1, ATG101, SERPINA3)	2 (DBH, BRSK2)
Cluster 2	72	41	4 (STAR, ATG2A, WNT11, NAE1)	1 (PPP3R1)
Cluster 3	68	85	4 (NDUFS5, TLR4, VDAC2, PSMC1)	2 (HRAS, TUBB4A)
Cluster 6	120	51	5 (SLC39A10, TUBB8, PSMD9, FZD2, MX1)	5 (PPP2R2A, NRBF2, CLUAP1, WNT9B, GGA3)
Subtype-unaware	145	124	7 (PSMA5, UBQLN1, CD44, SERPINA1, SERPINA3, TUBB3, TUBB6)	5 (UNC5C, LYN, DPYS, MAPK8IP1, SLC19A1)
Sum of unique genes in clusters	411	275	21	11

Twenty-one unique AD-related promoter regions were found to have increased accessibility, and 11 unique AD-related promoter regions were found to have decreased accessibility during AD. Increased promoter accessibility of SERPINA3 and PSMA5 was also found in the overall AD phenotype, and decreased promoter accessibility of SLC19A1 was also found in the overall AD phenotype. Interestingly, the remaining AD-related differentially accessible regions identified from each cluster are unique.

### Multi-omic Integrative Analyses Revealed Genes That Exhibit Alterations in Both Promoter Accessibility and Gene Expression

**Fig. 5:**
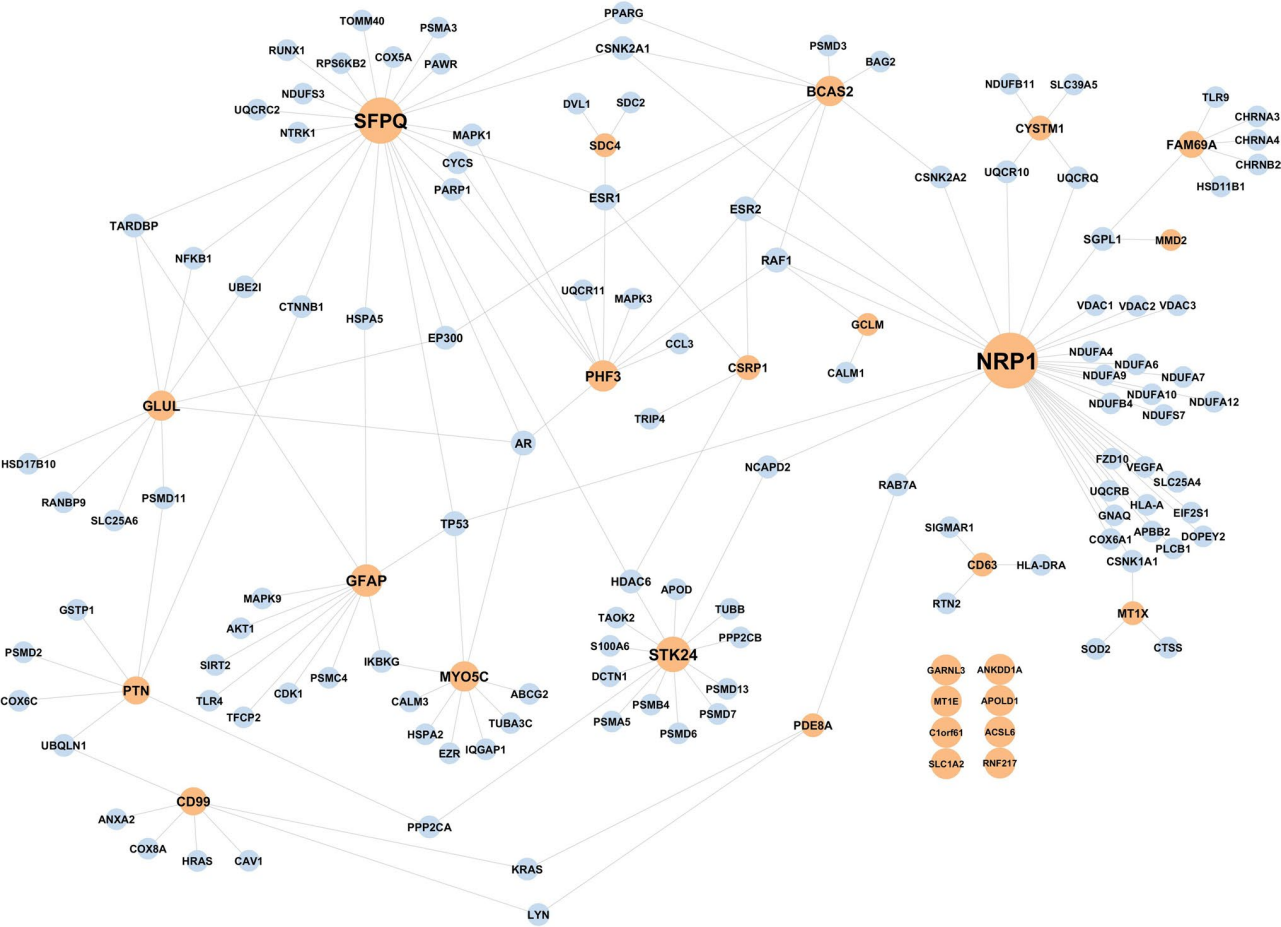
Genes that exhibit changes in both promoter accessibility and gene expression were found to be interacting with AD-related genes in the PPI network. Orange and blue colours correspond to genes with altered promoter accessibility/gene expression and AD-related genes, respectively

After identifying differentially accessible promoter regions in each astrocyte cluster, these findings were compared with the DEGs identified in each cluster. We identified 29 genes that exhibited changes in both promoter accessibility and gene expression. Most of these genes were observed to be strongly associated with the AD-related genes in the PPI network (Fig. 5). NRP1, SFPQ, and STK24 are the genes that interact most with the AD genes.

Although promoter accessibility and gene expression were found to be altered in the same direction for certain genes, an inverse relation was also observed. Table 3 shows certain genes that exhibit change in promoter accessibility and gene expression, and they were found to be strongly associated with astrocytic contribution to AD.

**Table 3:** The list of genes exhibiting changes in both promoter accessibility and gene expression in the same cluster during AD and possible transcriptional regulators of these genes in given clusters. “Upregulated TFs” and “Downregulated TFs” columns represent the up and downregulated transcription factors in the given clusters based on snRNA-seq data, and these transcription factors were identified to be capable of binding to the promoter regions of the corresponding genes based on our motif analysis results. Asterisk (*) refers to the TFs that were previously reported to regulate corresponding genes based on the information taken from the hTFtarget database, which relies on ChIP-seq data

	Genes	Gene expression	Chromatin accessibility	TFs that have motifs in gene promoter	
				Upregulated TF**s**	Downregulated TFs
Cluster 0	GARNL3	Downregulated	Decreased	ATF3*, ZNF680, FOS*	HES5
	SLC1A2	Downregulated	Increased	ATF3*, BCL6, PBX3*, RFX2	SOX9
	GCLM	Upregulated	Decreased	ZSCAN31, PBX3*, NR4A1, FOS*, RFX2*	
	CHI3L1	Upregulated	Decreased	RARB, ZSCAN31, FOXK1, ZNF680, PBX3*, FOS*	
Cluster 1	PTN	Downregulated	Increased	ATF3*, FOS*, TGIF1	
Cluster 3	GLUL	Downregulated	Decreased	NR4A2, NR1D2, THRB, RARB, ZNF680, HMBOX1, MXI1*, RFX2, ZNF331	JUN, NFIA, STAT1*, ETV1*, PKNOX2, HIF1A, FOS*, MEIS2, SOX9, JUNB
Cluster 6	SLC6 A11	Downregulated	Decreased		
	SDC4	Downregulated	Increased		

The contrast between gene expression and chromatin accessibility may be caused by many different mechanisms. Here, due to the existence of chromatin accessibility data, we focused on the TFs since they are capable of binding to promoter regions of genes of interests. Particularly, we focused on the TFs that are differentially up and downregulated in the given clusters during AD. In order to determine the possible TF regulators of the gene of interest, motif analysis was conducted. We supported our motif analysis results with the ChIP-seq results taken from a comprehensive human transcription factor database, hTFtarget, [40], which contains thousands of human ChIP-seq datasets from various tissues and reports the exact binding of TFs to gene promoter regions.

GTPase Activating Rap/RanGAP Domain Like 3 (GARNL3) gene was found to be upregulated in various brain regions during AD [41]. In our results, GARNL3 gene was downregulated during AD in all clusters, except for clusters 3 and 6, and its chromatin accessibility was significantly reduced in cluster- 0 AD astrocytes. Cell-specific regulation of GARNL3 during AD has not yet been completely enlightened, and potential roles and regulators of GANRNL3 in AD might be important for disease pathology. ATF3, ZNF680, FOS, and HES5 TFs were shown to regulate the transcriptional activity of GARNL3 based on our motif analysis results. ATF3 and FOS were previously reported to bind to GARNL3 promoter in the hTFtarget database, thus validating our results. SLC1A2 is mainly expressed by astrocytes in CNS and was reported to regulate glutamate metabolism [42], and its downregulation might reduce neurotransmitter homeostasis in CNS. A recent post-mortem human brain study proposed that high SLC1A2 expression in astrocytes during AD was neuroprotective [43]. SLC1A2 was downregulated in AD astrocytes in all clusters in our results, thereby might indicate the loss of protective functions. Interestingly, the promoter accessibility of SLC1A2 was found to be increased in cluster 0, and this situation might be caused by the binding of a repressor TF to the SLC1A2 promoter. In our motif analyses, we found that ATF3, BCL6, PBX3, RFX2, and SOX9 might regulate the transcription of SLC1A2 as they can bind to its promoter. ATF3 and PBX3 were reported to regulate SLC1A2 in the hTFtarget database. CHI3L1 encodes for a protein called YKL-40, a CSF biomarker for AD [44] and associated with astrocyte reactivity and neuroinflammation [45]. Its expression has been proposed to suppress phagocytic activation of glial cells and promote Aβ accumulation [44]. CHI3L1 was upregulated in AD astrocytes in our results in all clusters except for cluster 7, thereby might be neurotoxic. On the other hand, its promoter accessibility was shown to be reduced significantly in cluster- 0 AD astrocytes, and this behaviour might be neuroprotective. Our motif analyses showed that RARB, ZSCAN31, FOXK1, ZNF680, PBX3, and FOS TFs can bind to CHI3L1 promoter and might be its possible regulators. SDC4 was reported to be the astrocyte-specific syndecan [9]. It was proposed that SDC3 and SDC4 might be an important target for Aβ to enter inside the cells, and overexpression of SDC3 and SDC4 can contribute to the uptake and fibrillation of Aβ [46]. SDC4 was found to be downregulated in AD astrocytes in all clusters and overall AD astrocyte phenotype. Thereby, this mechanism might be leading to reduced uptake of Aβ. On the other hand, chromatin accessibility of SDC4 was found to be increased in cluster- 6 AD astrocytes. SDC4-mediated internalization of Aβ by astrocytes might be investigated for potential therapeutic roles for AD.

### PTN Might Be Regulated by ATF3 Transcription Factor in Cluster 1

**Fig. 6:**
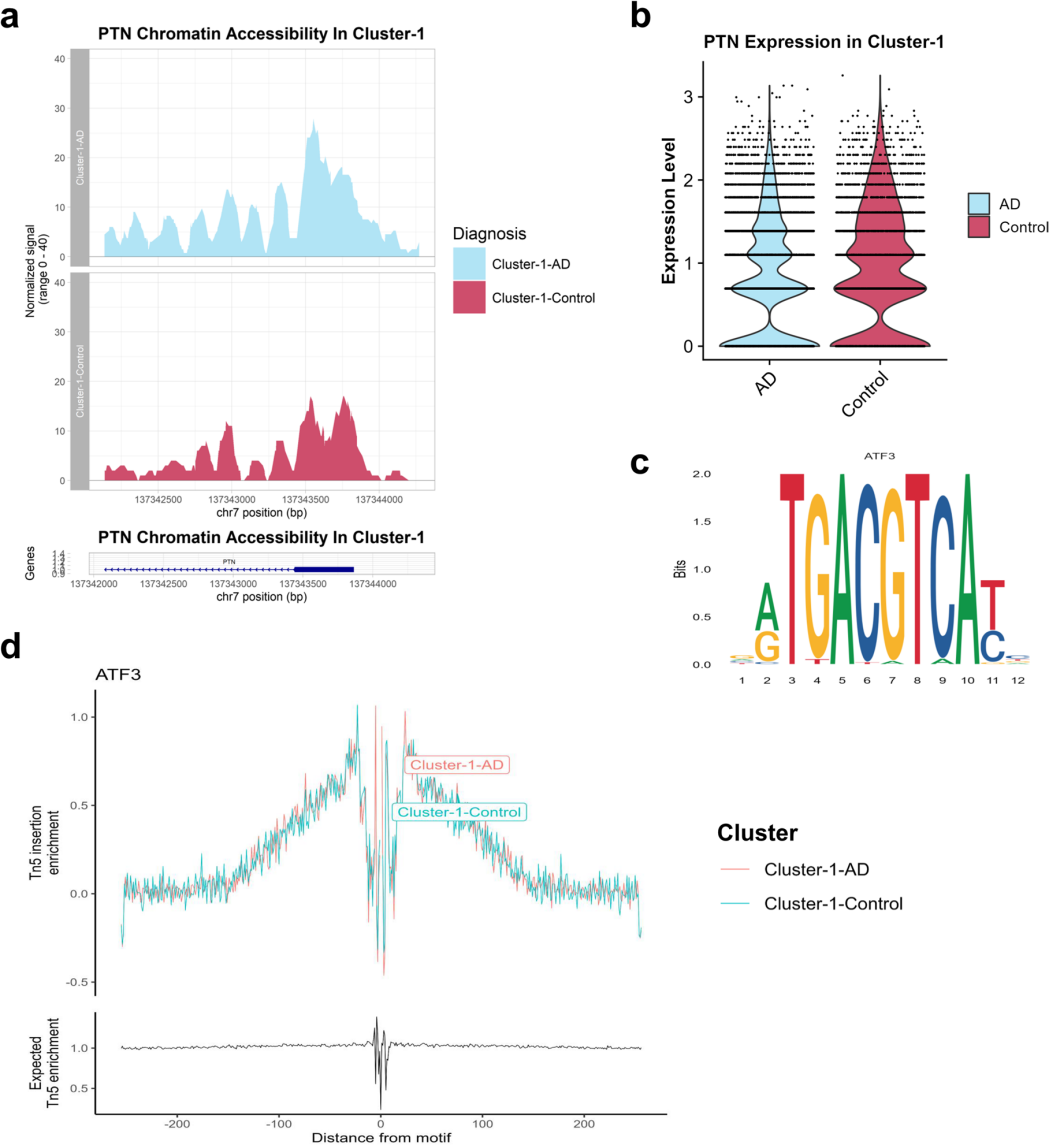
PTN might be regulated by ATF3 transcription factor. **a** Promoter accessibility of PTN gene in cluster 1. **b** PTN gene expression in cluster 1. **c** ATF3 TF motif. **d** Motif footprinting result shows that ATF3 binds to certain gene promoters in cluster 1

PTN is a cytokine expressed in the human brain, and its expression level was shown to be increased in response to inflammatory triggers that might be caused by neurodegenerative diseases [47]. In a recent study, it has been proposed that PTN might be an important regulator in neuroinflammation [47]. PTN was reported to be upregulated in AD [48]. However, in the entorhinal cortex and superior frontal gyrus astrocytes, it was found to be downregulated [49]. PTN upregulation in multiple sclerosis astrocytes was shown to be neuroprotective as PTN was shown to reduce proinflammatory signalling in astrocytes and promote neuronal survival [50]. In our case, PTN gene was found to be downregulated (fold-change 0.76 and adjusted *p*-value 1.34 × 10^−40^) in cluster- 1 AD astrocytes (Fig. 6b). Downregulation of PTN might considered to be a neurotoxic behaviour. On the other hand, its promoter accessibility was found to be significantly increased (fold-change 1.93 and *p*-value 0.0013) in cluster- 1 AD astrocytes (Fig. 6a).

Increased promoter accessibility of PTN in cluster 1 might be associated with the regulation of PTN in cluster 1 by TFs. We searched for possible transcription factors that might regulate PTN expression. We found that ATF3, FOS, and TGIF transcription factors are upregulated in cluster 1 during AD based on our snRNA-seq data. According to motif analysis results, ATF3 and FOS transcription factors were shown to be able to bind to PTN promoter. The accuracy of the motif analysis results was supported with the hTFtarget database as they also reported that ATF3 can bind to PTN promoter. Also, motif footprinting analysis was performed for ATF3 TF, and it was validated that it binds to certain gene promoters in cluster 1 (Fig. 6c, d). As it can be seen from Fig. 6d, Tn5 insertion event is increasing up to some point, then starts to decrease as it approaches to the centre of the motif due to TF existence in the promoter region. Eventually, PTN expression was obtained to be downregulated in cluster- 1 AD astrocytes, and this might be considered neurotoxic. Increased chromatin accessibility of PTN in cluster 1 might be referring to neuroprotective behaviour in the epigenetic level. But this behaviour does not seem to appear in the mRNA level.

## Discussion

Despite the fact that AD was considered to be a neuron-based disorder, recent studies have shown that the contribution of glial cells to AD pathology is undeniable. Due to the conflicting behaviours of astrocytes during AD, their behaviours require in-depth analysis. For this purpose, astrocytes obtained from the comprehensive single-nuclei transcriptome and epigenome datasets for AD, which were generated from post-mortem human brain prefrontal cortex [10, 11], were re-analyzed in this study to identify astrocyte-subtype-specific transcriptomic and epigenetic responses to AD.

Our single-nuclei-based data integration and network-mapping approaches were proven to capture disease-related genes with a higher sensitivity compared to the Sadick study [10] and enabled several unique findings not reported in the studies that generated the data used in this study [10, 11]. We were able to identify cluster-specific AD-related genes (PAR- 4, PPARA, FDPS, LDLR, TLR4, GBP2, PI3 K, PLCB1, S100 A6, ITPR1, NQO1, ECE1) and several enriched molecular pathways (learning or memory, regulation of mitochondrial membrane potential, negative regulation of release of cytochrome c from mitochondria, ferritin complex, calmodulin binding, response to calcium ion, alpha-linolenic acid metabolic process, gamma-aminobutyric acid transport, regulation of synapse structure or activity, astrocyte end-foot, neurotransmitter receptor activity, transmitter-gated channel activity, neurofibrillary tangle, amyloid beta metabolic process, amyloid beta formation, tau protein binding) that were not reported in the original studies [10, 11].

The identified altered biological processes (Fig. 3) were associated with several genes with previously reported astrocytic roles. For example, PAR-4 was found to be expressed by apoptotic astrocytes that surround amyloid plaques [51], and we found that PAR-4 was uniquely upregulated in cluster- 1 AD astrocytes. PPARA agonists were shown to improve autophagy in astrocytes and improve memory impairment in mice [52]. PPARA was found to be uniquely upregulated in cluster- 8 AD astrocytes in our results. Therefore, those astrocytes might be neuroprotective. Astrocytes are responsible for cholesterol production and delivery [53], and farnesyl diphosphate synthase (FDPS) gene encodes for an enzyme that has a role in cholesterol synthesis [54]. FDPS was found to be uniquely downregulated in cluster- 1 AD astrocytes. ABCA1 is a transporter that plays a vital role in maintaining stable levels of cholesterol in the brain since it regulates ApoE levels [53]. It was found to be upregulated in all AD astrocyte clusters, except clusters 3 and 4, implying a neuroprotective role. Low-density lipoprotein receptor (LDLR) controls the abundance of ApoE in the brain, and by this way, it controls the uptake and degradation of Aβ by astrocytes [55]. It was found to be uniquely downregulated in cluster- 3 AD astrocytes. Since this might reduce the astrocytic Aβ uptake and clearance, it can be neurotoxic. Clusterin (CLU) is a well-known LOAD gene related with cholesterol, and it was previously reported to be upregulated in human AD astrocytes [56]. It was upregulated in all AD astrocytes except for cluster 3, implying a neuroprotective behaviour.

We also identified several genes associated with the immune-related pathways (Fig. 3). Aβ was shown to bind to astrocytic TLR4, and its activation was shown to improve the clearance of amyloid plaques in AD mouse models [57–60]. TLR4 was uniquely downregulated in cluster- 1 AD astrocytes in our results. CD44 is a cell surface glycoprotein involved in inflammation and can be found in astrocytes [61, 62]. The number of CD44-positive astrocytes was shown to increase in the brains of AD patients [61]. CD44 was found to be upregulated in cluster- 0, 1, 2, 4, 6, and 8 AD astrocytes in our results. Similar to CD44, guanylate-binding protein 2 (GBP2) was considered to be an inflammatory reactive astrocyte marker [33], and it was uniquely upregulated in cluster- 1 AD astrocytes in our results. Another gene involved in inflammation is SERPINA3 [29]. Studies have shown that SERPINA3 is mainly expressed by reactive astrocytes that surround amyloid plaques [63]. SERPINA3 was found to be upregulated in cluster- 0, 2, 4, 5, and 6 AD astrocytes in our results; therefore, those clusters might be promoting the Aβ accumulation. Another crucial signalling pathway is phosphatidylinositol 3-kinase (PI3K), and it is involved in inflammation, maintenance of synaptic plasticity, and long-term potentiation, which was shown to be associated with memory [64, 65]. It was found that PI3K and AKT levels were increased in reactive astrocytes [66]. Similarly, in our results, PI3K was found to be upregulated in cluster- 2, 3, and 5 AD astrocytes. Additionally, AKT3 was found to be uniquely upregulated in cluster- 3 AD astrocytes.

Iron overload and calcium overload have been reported to be crucial in AD. Solute Carrier Family 39 Member 14 (SLC39A14) is a transmembrane transporter expressed by astrocytes that regulates the uptake of iron [67, 68]. Previously, reactive astrocytes were shown to have high iron uptake compared to resting astrocytes [69, 70]. SLC39A14 was found to be uniquely upregulated in cluster 1 in our study, and this may cause neurotoxicity as it might lead to iron overload. Phospholipase C-β1 (PLCB1) gene was proposed to contribute to AD pathology through calcium overload [71]. We found that PLCB1 is downregulated in all AD astrocyte clusters except for clusters 0, 3, and 8. Downregulation of PLCB1 might lead to the alleviation of calcium overload, thereby might be neuroprotective. S100 calcium-binding protein A6 (S100A6) is a calcium-binding protein highly expressed by astrocytes in AD around amyloid plaques, and it can lead to Aβ degradation [72]. S100A6 was found to be upregulated in cluster- 1, 2, and 3 AD astrocytes in our results, implying a neuroprotective behaviour. Inositol 1,4,5-trisphosphate receptor type 1 (ITPR1) is another gene related with calcium signalling pathway and can contribute to AD pathology [73]. ITPR1 gene was found to be uniquely downregulated in cluster- 3 AD astrocytes. Heat shock proteins HSP90AA1 and HSP90AB1 are chaperons associated with unfolded protein binding [74]. While HSP90AB1 is expressed by healthy and AD astrocytes, HSP90AA1 is expressed only by AD astrocytes [74]. HSP90AA1 was found to be upregulated in AD astrocytes in all clusters in our results, while HSP90AB1 was found to be upregulated in cluster- 0, 1, 2, 4, 5, and 6 AD astrocytes. As chaperons are associated with protein folding, their upregulation might be related with neuroprotection. ITM2B and ITM2C were previously reported to be upregulated in human reactive astrocytes in AD [45]. They also encode a chaperone protein that was previously shown to decrease Aβ formation in the human AD brain [75]. We found that both ITM2B and ITM2C were upregulated in cluster- 2 AD astrocytes. Therefore, their ability to limit Aβ accumulation might be neuroprotective.

Response to oxidative stress is another biological process identified to be altered (Fig. 3). Superoxide dismutase 2 (SOD2) is an antioxidant enzyme that catalyzes the conversion of superoxide into hydrogen peroxide [76]. SOD2 overexpression was shown to reduce amyloid plaques [77] as a response to oxidative stress. SOD2 was shown to be upregulated in cluster- 0, 1, 2, 3, 4, 5, and 6 AD astrocytes. Therefore, its role can be considered to be neuroprotective. MT2A (metallothionein- 2) is another antioxidant molecule produced by astrocytes and is considered a neuroprotective response of astrocytes against oxidative stress [78]. It was found to be uniquely enriched in cluster- 2 and significantly upregulated in cluster- 0, 4, 5, 7, and 8 AD astrocytes in our results. NAD(P)H quinone dehydrogenase 1 (NQO1) was expressed by astrocytes that surround amyloid plaques, and the upregulation of NQO1 has been proposed to be neuroprotective [79]. We also found that NQO1 was upregulated in cluster- 0, 2, and 3 AD astrocytes.

Astrocytes were shown to produce enzymes that can degrade Aβ into smaller fragments and might reduce the amyloid burden in the brain. One of these enzymes is called endothelin-converting enzyme 1 (ECE1) [80]. ECE1 was found to be upregulated in cluster- 0, 1, 3, and 5 AD astrocytes, implying neuroprotective behaviour. TMED10 is a transmembrane protein, and decreased TMED10 expression leads to increased γ-secretase cleavage of APP [81]. TMED10 was found to be uniquely downregulated in cluster- 3 AD astrocytes. Therefore, it might be contributing to increased amyloid burden in AD brain. Mitogen-activated protein kinase 10 (MAPK10) is a kinase that is expressed by astrocytes [82], and it was found to enhance the production of Aβ and lead to the formation of NFTs [39]. We found that MAPK10 is uniquely upregulated in cluster- 3 AD astrocytes and might be a contributor to the neurotoxicity in AD. In AD, intraneuronal accumulation of tau protein is a well-known phenomenon, but microtubule-associated protein tau (MAPT) was shown to be also expressed by astrocytes [31]. It was shown that Aβ binds to the calcium-sensing receptor in human astrocytes, leading to the production and release of phosphorylated tau [31]. Thereby, astrocytes that encounter with Aβ might produce hyperphosphorylated tau and release it. MAPT was upregulated in cluster- 3, 6, and 8 AD astrocytes in our results, and this behaviour might be considered neurotoxic. Another element involved in amyloid beta clearance is aquaporin 4 (AQP4), which has been reported to be also involved in amyloid beta and tau clearance [83–85]. It is mainly expressed by perivascular end-feet of astrocytes [86]. AQP4 deletion was shown to increase amyloid accumulation in 5xFAD mice cerebral cortex [87]. AQP4 was found to be uniquely upregulated in cluster- 2 AD astrocytes, which might be considered a neuroprotective response.

Moreover, we also tried to provide a detailed characterization of A1 reactive astrocytes for driving neurotoxicity by highlighting uniquely up and downregulated genes in cluster 3, which were not addressed in the original studies [10, 11]. Some uniquely expressed transcripts in the cluster- 3 AD astrocytes might be enhancing the AD pathology. The downregulated subnetwork of cluster 3 included GLUL, LDLR, and TMED10 genes, which were uniquely downregulated in cluster 3 during AD and might be causing neurotoxicity. We also identified that TRIM25 and TRIM67 were interacting with these genes, and for this reason, these TRIM E3 ubiquitin ligase members might be contributing to A1 reactive phenotype to modulate AD pathology. The upregulated subnetwork of cluster 3 included MAPK10 and MAPT genes, which might be also crucial for A1 reactive phenotype in AD.

In addition, by the analysis of the snATAC-seq data for the same tissue, we were able to identify the genes (PSMA5, PAICS, SLC19A1, NTRK1, TP63, IL12B, OLR1, ATG101, DBH, BRSK2, STAR, ATG2A, WNT11, NAE1, PPP3R1, NDUFS5, TLR4, VDAC2, PSMC1, HRAS, TUBB4A, SLC39A10, TUBB8, PSMD9, FZD2, PPP2R2A, NRBF2, CLUAP1, WNT9B, GGA3, UBQLN1, SERPINA1, TUBB3, TUBB6, UNC5C, DPYS, MAPK8IP1, SLC19A1) reported in Table 2 that exhibited changes in promoter accessibility and associated with AD, and these genes were not reported in the original studies [10, 11].

In addition, we were also able to identify genes that exhibited changes in both promoter accessibility and gene expression in their respective clusters (Fig. 5). For some of these genes (BCAS2, CYSTM1, FAM69 A, PHF3, STK24, ANKDD1 A, RNF217), not much information was available in the literature from the AD perspective, and these might be novel genes for the disease pathology.

Lastly, the regulation of PTN gene by ATF3 transcription factor, which seems to be closely associated with the AD pathology, was a unique finding of our study; it was not discussed in either studies. PTN gene in cluster 1 was predicted to be regulated by ATF3 TF. ATF3 (activating transcription factor 3) is one of the members of the ATF/cAMP responsive element-binding (CREB) protein family. Many studies have shown that ATF3 contributes to stress and neuroinflammation, as comprehensively reviewed by Heinrich et. al (2024), and hence, it is hypothesized to be involved in AD pathogenesis. In one of the studies, ATF3 was found to bind to the CCL4 promoter and regulate its transcription to modulate amyloid deposition and, hence, modulate AD pathology in mice [88]. In that study, ATF3 expression displayed a positive correlation with CCL4 [88]. Despite the fact that the name ATF3 refers to activating transcription factor, it was also reported to act as a repressor [89]. Interestingly, it was shown to repress the expression of CCL4 in murine macrophages in a study [90]. Therefore, we cannot clearly classify ATF3 transcription factor as an activator or repressor [91]. Moreover, ATF3 could be considered as a marker for neuronal injury during AD [92, 93]. PTN and ATF3 TF interaction was previously shown through ChIP-seq, validating the results of this study. The downregulation of PTN gene was previously reported to cause the loss of astrocytic neuroprotective functions [50], and as it was found to be downregulated in cluster 1, this behaviour might correspond to the same phenomenon.

In this study, we focused on two single-nuclei AD datasets from the human prefrontal cortex, where one primarily enabled the detailed analysis of astrocyte subtypes since it included a considerably high number of astrocyte nuclei, and the other enabled the incorporation of epigenomic data from the exact same tissue into our subtype-specific analysis. We analyzed these two datasets independently. Regarding dataset integration, although an ideal approach would be to integrate the two datasets first and then subset astrocytes, this was not possible due to the computational limitations of the packages used in this study. Instead, we ensured that both datasets had a homogenous distribution of the individuals within the clusters. On the other hand, the availability of algorithms that will enable the integration of multiple datasets at first will minimize errors that may arise from differences in preprocessing pipelines. Unlike the recent single-nucleus multiome sequencing approach [94], where both gene expression and chromatin accessibility can simultaneously be profiled from the same nuclei, we had to integrate independently generated snRNA-seq and snATAC-seq datasets, requiring intensive computational efforts. This approach posed some limitations such as the inability to map all nine snRNA-seq-based astrocyte subtypes onto the snATAC-seq space. Considering the substantial increase in the number of single-nucleus multiome sequencing datasets, these limitations will be overcome in future studies, leading to a clearer picture of the molecular mechanisms of astrocyte subtypes in AD.

## Supplementary Information

Below is the link to the electronic supplementary material.

Supplementary file1 (ZIP 122312 KB)

## Data Availability

The datasets utilized in this study, identified by accession numbers GSE167494 and GSE174367, were retrieved from the Gene Expression Omnibus (GEO) database. No new datasets were generated during the current study.
