# Supplementary material for: Functional Specificity of Astrocyte Subtypes in Alzheimer’s Disease: Decoding Disease Mechanisms Through Network-based Analysis of Integrated Single-Nuclei Multi-Omic Data
Source: Mol Neurobiol. 2025 Apr 29;62(9):11611–31. doi: 10.1007/s12035-025-04965-8 (PMC12367892; doi:10.1007/s12035-025-04965-8)
Supplement: Supplementary file 1 — Supplementary file1 (ZIP 122312 KB) [file 12035_2025_4965_MOESM1_ESM.zip › ESM_2.pdf]

Article title: Functional Specificity of Astrocyte Subtypes in Alzheimer's Disease: Decoding Disease Mechanisms through Network-based Analysis of Integrated Single-Nuclei Multi-Omic Data

Journal name: Molecular Neurobiology

Author names: Atılay İlgün, Tunahan Çakır

Author affiliations: Gebze Technical University, Department of Bioengineering, Gebze/Kocaeli/TÜRKİYE

Corresponding author: Tunahan Çakır (tcakir@gtu.edu.tr)

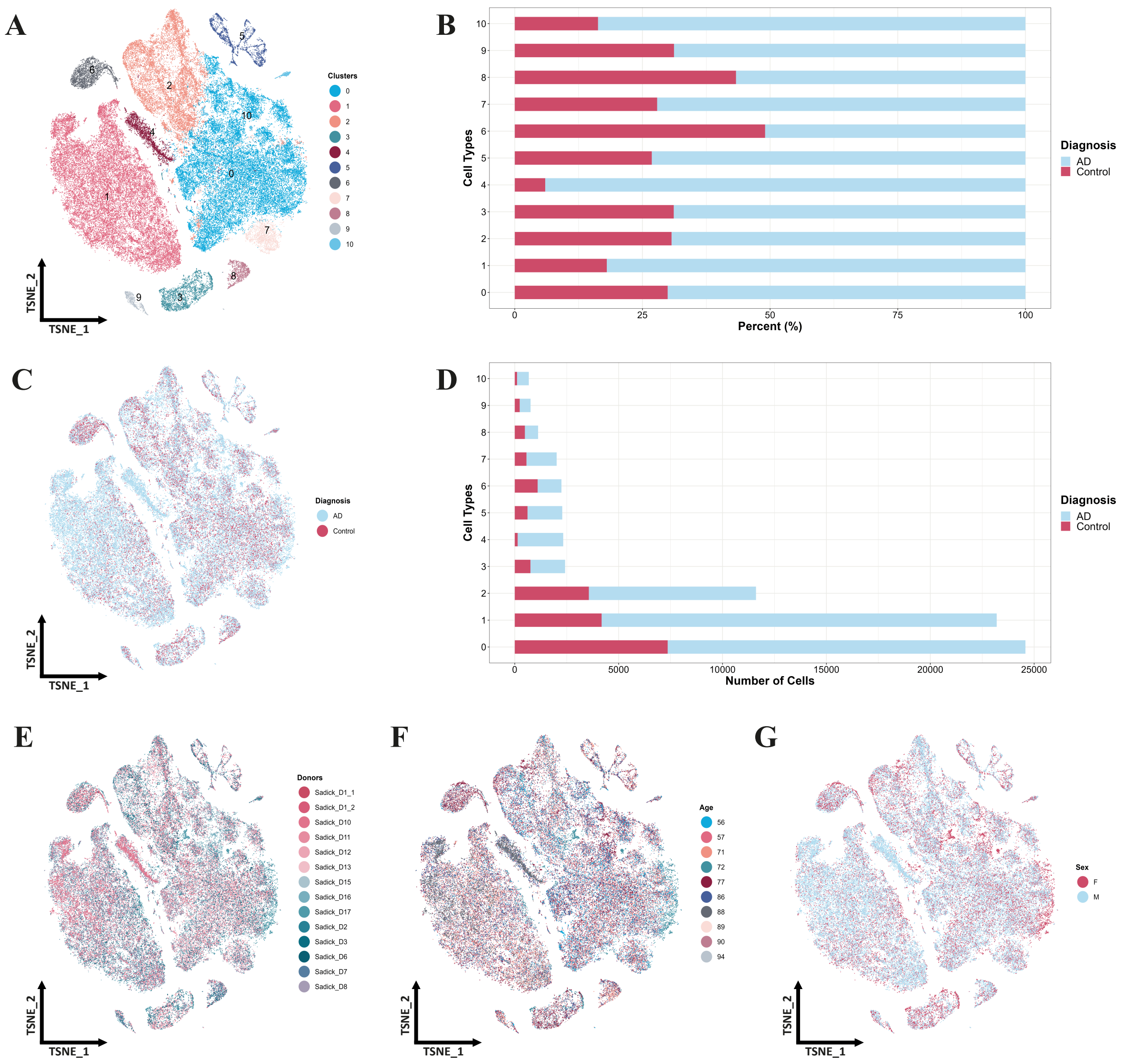

H

Cluster	Cell Number	AD	Control
0	24580	17220	7360
1	23197	19017	4180
2	11611	8046	3565
3	2422	1668	754
4	2331	2192	139
5	2285	1672	613
6	2246	1145	1101
7	2016	1454	562
8	1122	636	486
9	760	523	237
10	675	565	110
TOTAL	73245	54138	19107

I

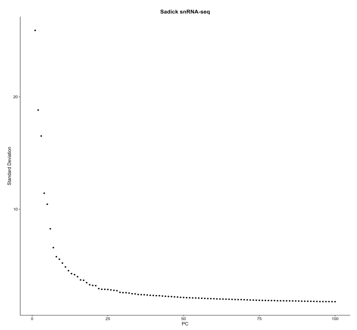

Online Resource-2A. Clustering of Sadick snRNA-seq dataset was validated by removing clusters driven by a single feature. (A) TSNE plot of whole CNS nuclei (73245 cells) in Sadick dataset . **(B)** Proportion of AD and control cases in each cluster. **(C)** TSNE plot generated to visualize the distribution of nuclei from whole CNS cells according to diagnosis. **(D, H)** Number of AD and control cells in each cluster. Cluster-4 is represented mostly by AD astrocytes (2192 AD and 139 control) and it was excluded for downstream analyzes. **(E-G)** TSNE plot generated to visualize the distribution of nuclei from whole CNS cells according to donors, donor ages and donor sexes respectively. **(I)** Elbow plot shows how many principle components should be used for downsteam analyzes.

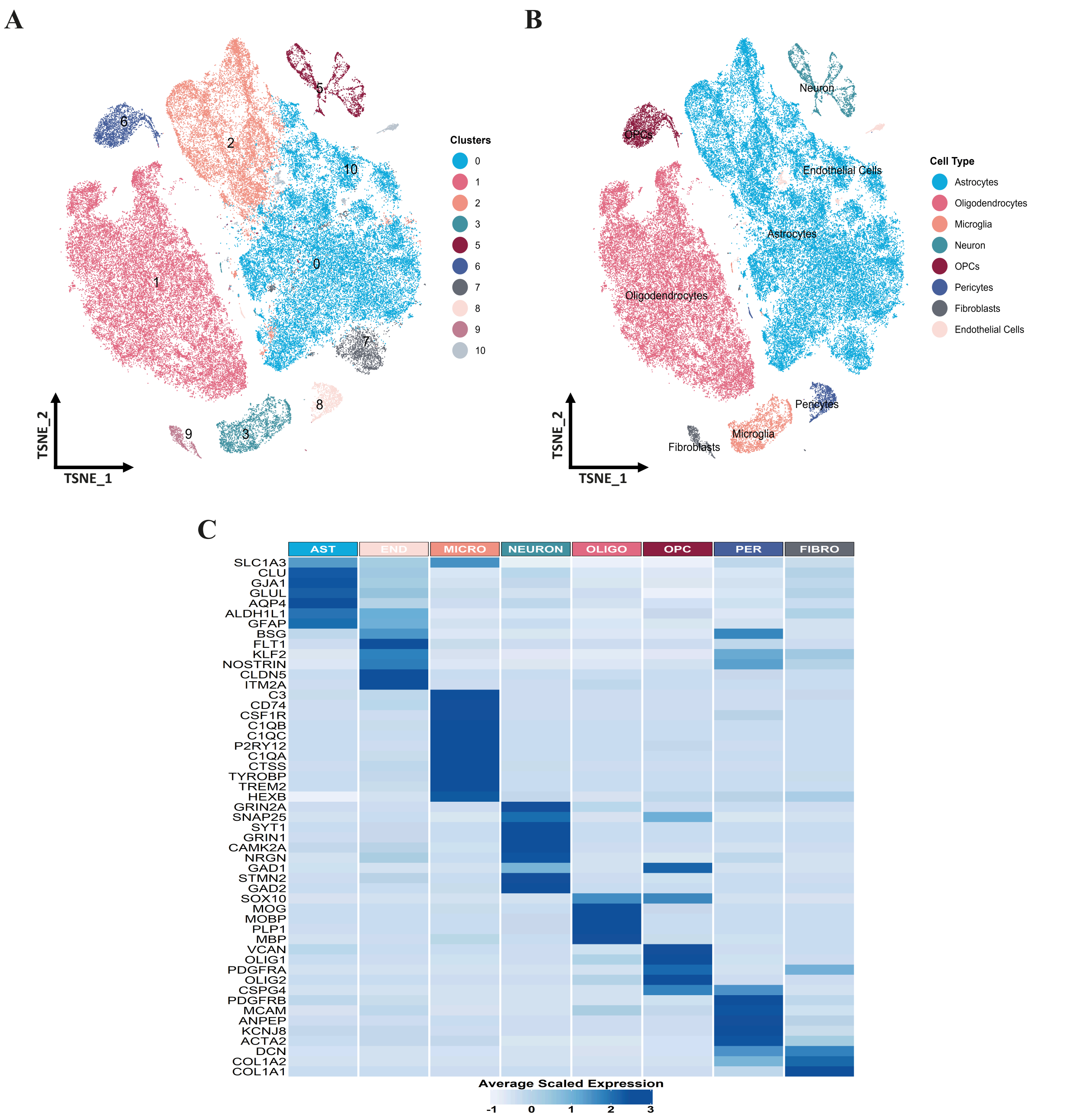

D

Cell Type	Cell Number	AD	Control
Astrocytes	38207	26720	11487
Oligodendrocytes	23197	19017	4180
Microglia	2422	1668	754
Neuron	2285	1672	613
OPCs	2246	1145	1101
Pericytes	1122	636	486
Fibroblasts	760	523	237
Endothelial Cells	675	565	110

Online Resource-2B. Sadick snRNA-seq dataset was annotated based on the differential expression of canonical cell type markers. **(A)** TSNE plot of whole CNS nuclei (70914 cells) in Sadick dataset after removal of cluster-4. **(B)** TSNE plot of annotated CNS. **(C)** Heatmap of canonical cell type markers used for annotation (AST: Astrocytes; END:Endotheli- al cells; MICRO: Microglia; OLIGO: Oligodendrocytes; OPC: Oligodendrocyte precursor cells; PER: Pericytes; FIBRO: Fibroblasts). **(D)** Number of AD and control cells in each cell type after annotation.

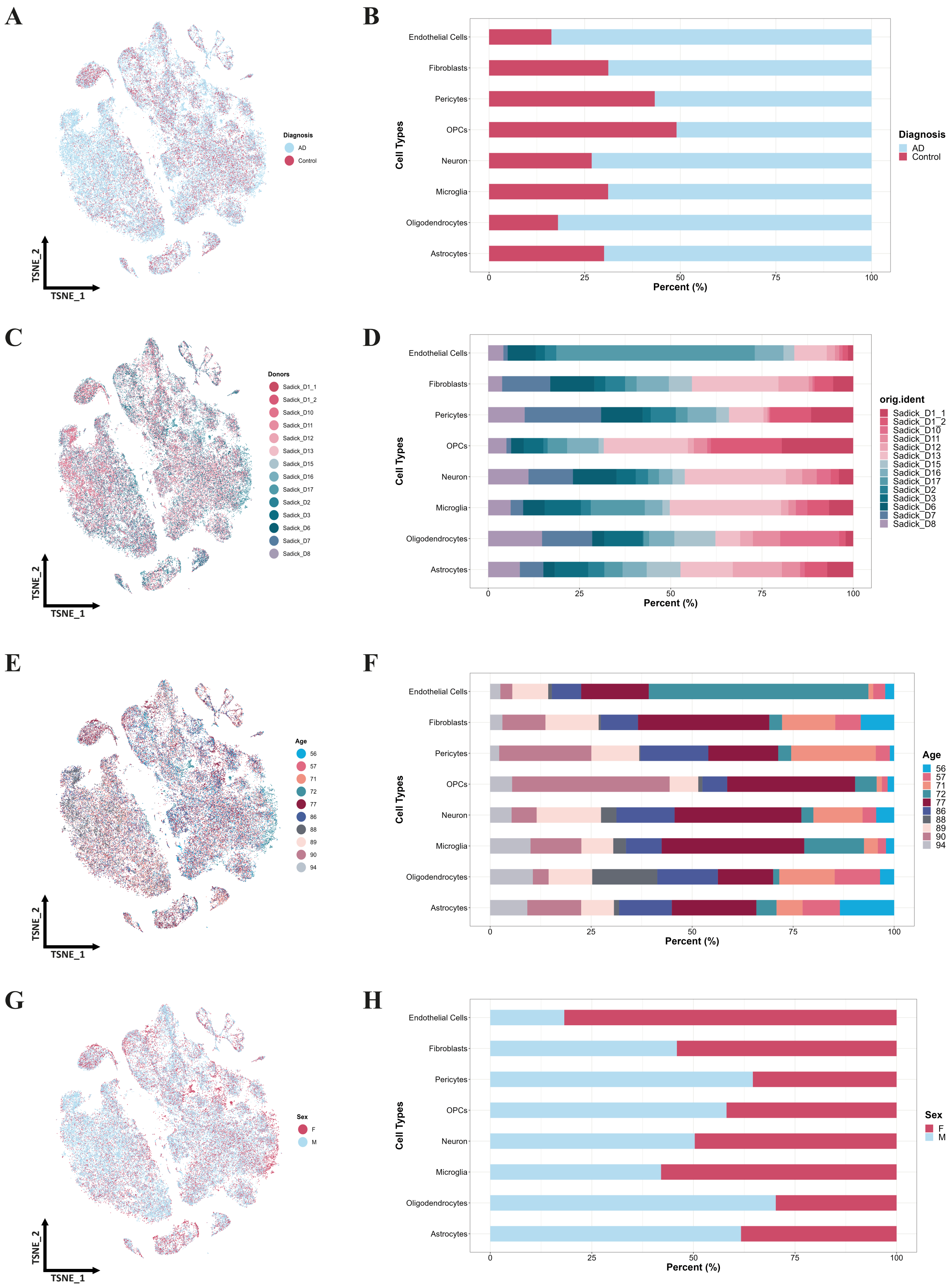

Online Resource-2C. Annotation of Sadick snRNA-seq dataset was validated by checking the distribution of certain features (diagnosis, donor distribution, donor age and sex) across cell types. (A-B) Distribution of cell types based on diagnosis. **(C-D)** Distribution of donors across cell types. **(E-F)** Distribution of donor ages across cell types. **(G-H)** Distributi- on of donor sexes across cell types.

A

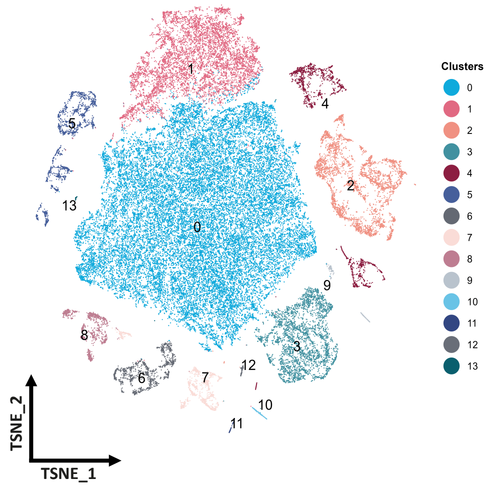

B

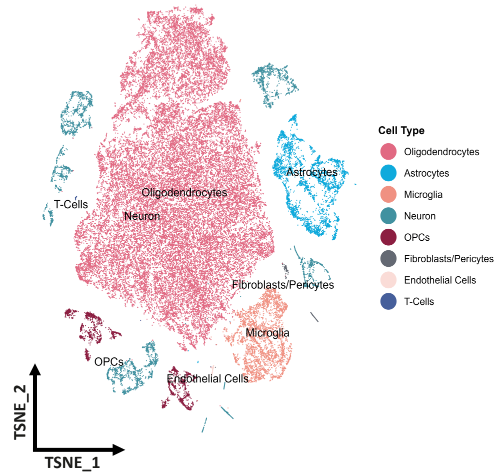

C

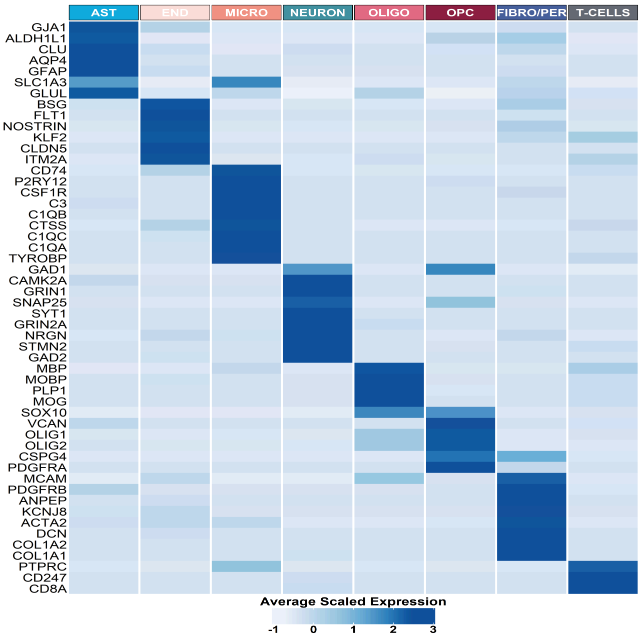

D

Cluster	Cell Number	AD	Control
0	29449	15737	13712
1	6633	5803	830
2	3813	2433	1380
3	3483	2315	1168
4	1775	1476	299
5	1649	1247	402
6	1252	923	329
7	1124	516	608
8	1032	870	162
9	284	186	98
10	168	125	43
11	127	53	74
12	122	76	46
13	73	51	22
TOTAL	50984	31811	19173

E

Cell Type	Cell Number	AD	Control
Oligodendrocytes	36082	21540	14542
Astrocytes	3813	2433	1380
Microglia	3483	2315	1168
Neuron	4971	3824	1147
OPCs	2156	1386	770
Fibroblasts/Pericytes	284	186	98
Endothelial Cells	122	76	46
T-Cells	73	51	22

F

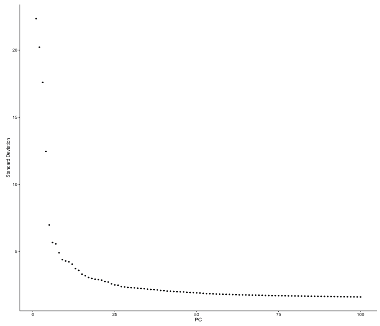

Online Resource-2D. Morabito snRNA-seq dataset was processed and annotated distribution annotated based on the differential expression of canonical cell type markers. **(A)** TSNE plot of whole CNS nuclei (50984 cells) in Morabito dataset. **(B)** TSNE plot of annotated CNS. **(C)** Heatmap of canonical cell type markers used for annotation (AST: Astrocytes; END:Endothelial cells; MICRO: Microglia; OLIGO: Oligodendrocytes; OPC: Oligodendrocy- te precursor cells; FIBRO/PER: Fibroblasts and pericytes). **(D)** Number of AD and control cells in each cluster. **(E)** Number of AD and control cells in each cell type after annotation. **(F)** Elbow plot shows how many principle components should be used for downsteam analyzes.

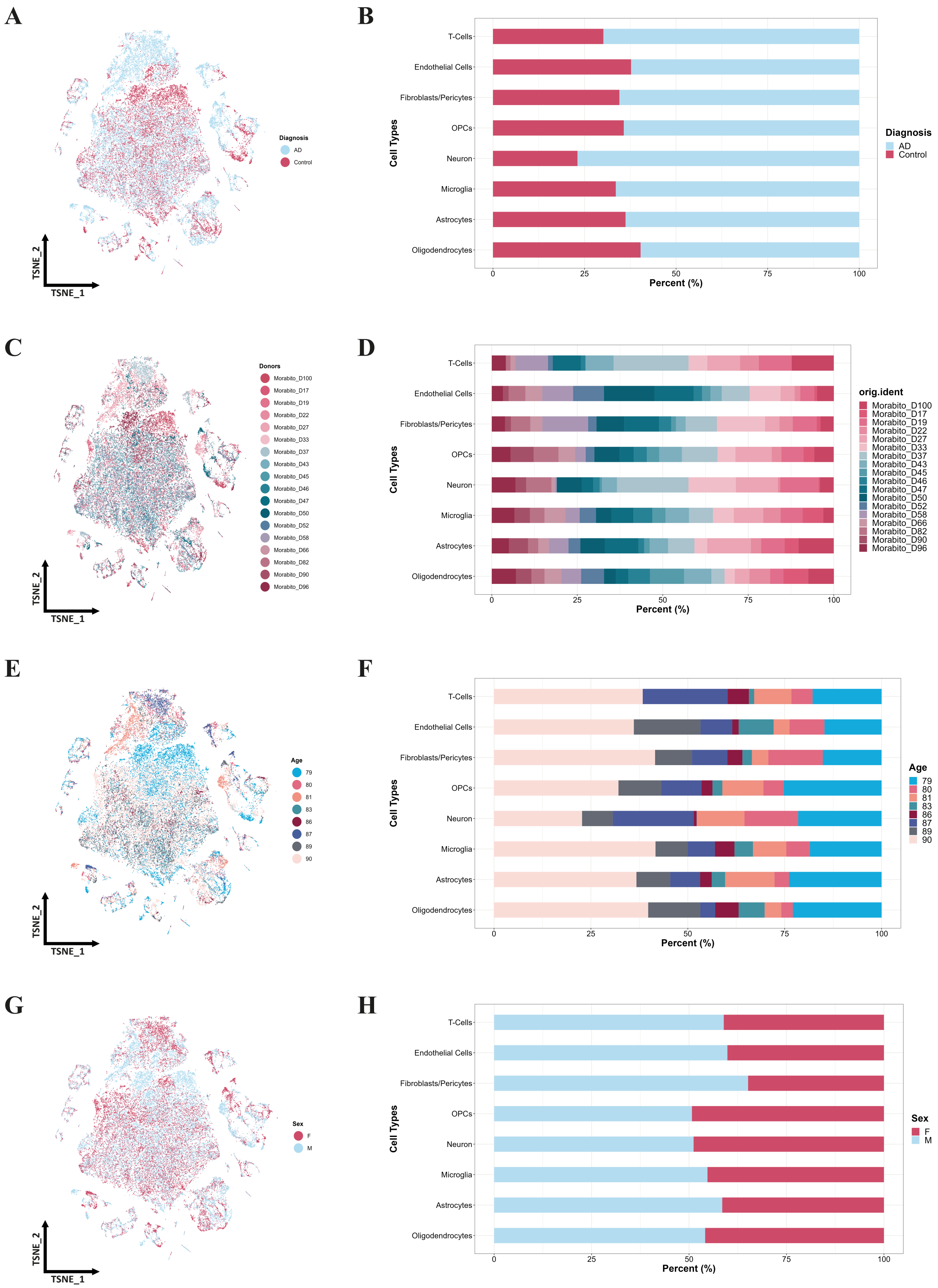

Online Resource-2E. Annotation of Morabito snRNA-seq dataset was validated by checking the distribution of certain features (diagnosis, donor distribution, donor age and sex) across cell types. (A-B) Distribution of cell types based on diagnosis. **(C-D)** Distribution of donors across cell types. **(E-F)** Distribution of donor ages across cell types. **(G-H)** Distributi- on of donor sexes across cell types.

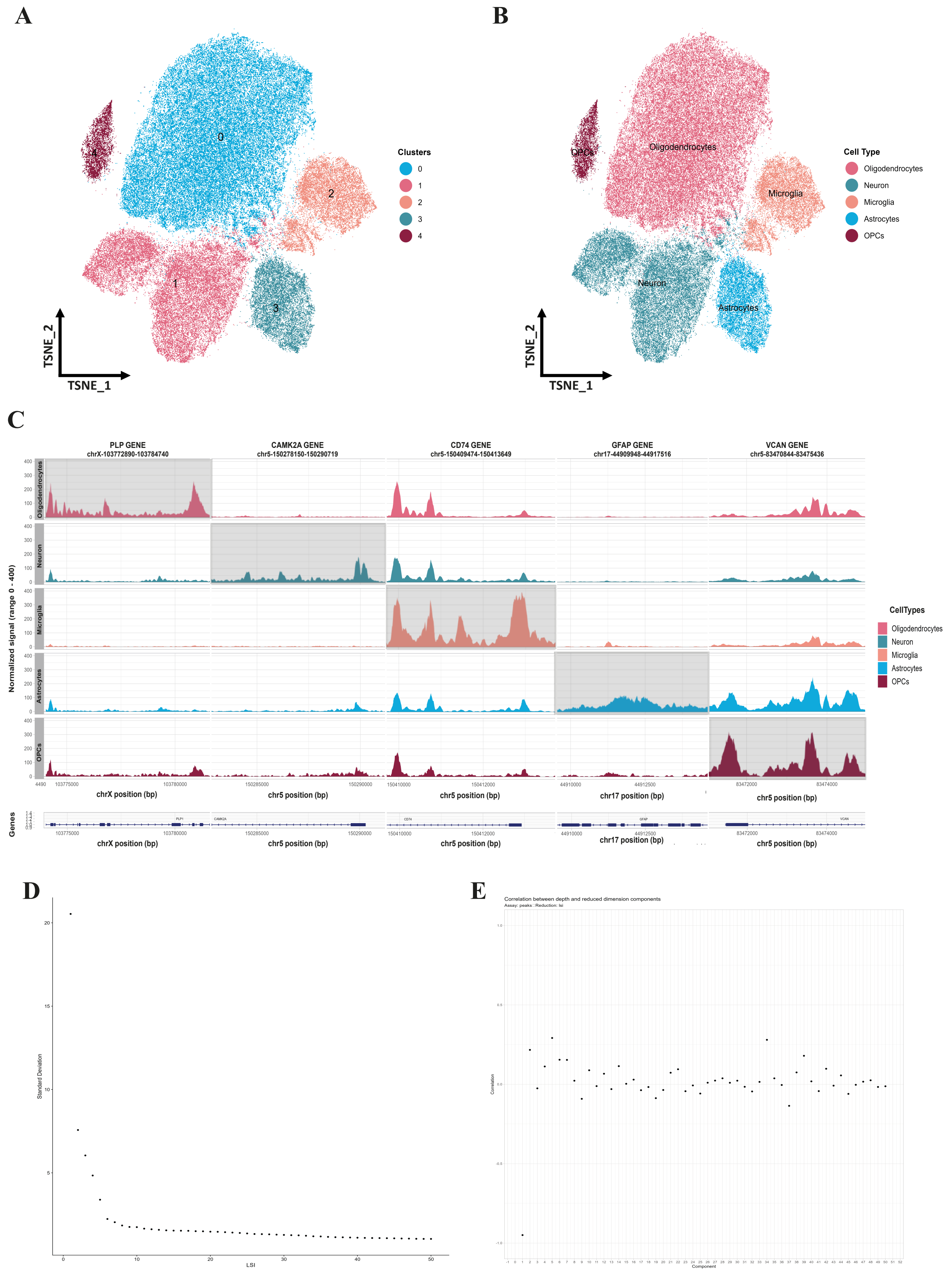

Online Resource-2F. Morabito snATAC-seq dataset was processed and annotated distribution annotated based on the differential accessibility of promoter regions of canonical cell type markers. **(A)** TSNE plot of whole CNS nuclei (101137 cells) in Morabito data- set. **(B)** TSNE plot of annotated CNS. **(C)** Promoter accessibility of canonical cell type markers used for annotation. **(D)** Elbow plot shows how many LSI components should be used for downsteam analyzes. **(E)** Correlation between LSI components and sequencing depth.

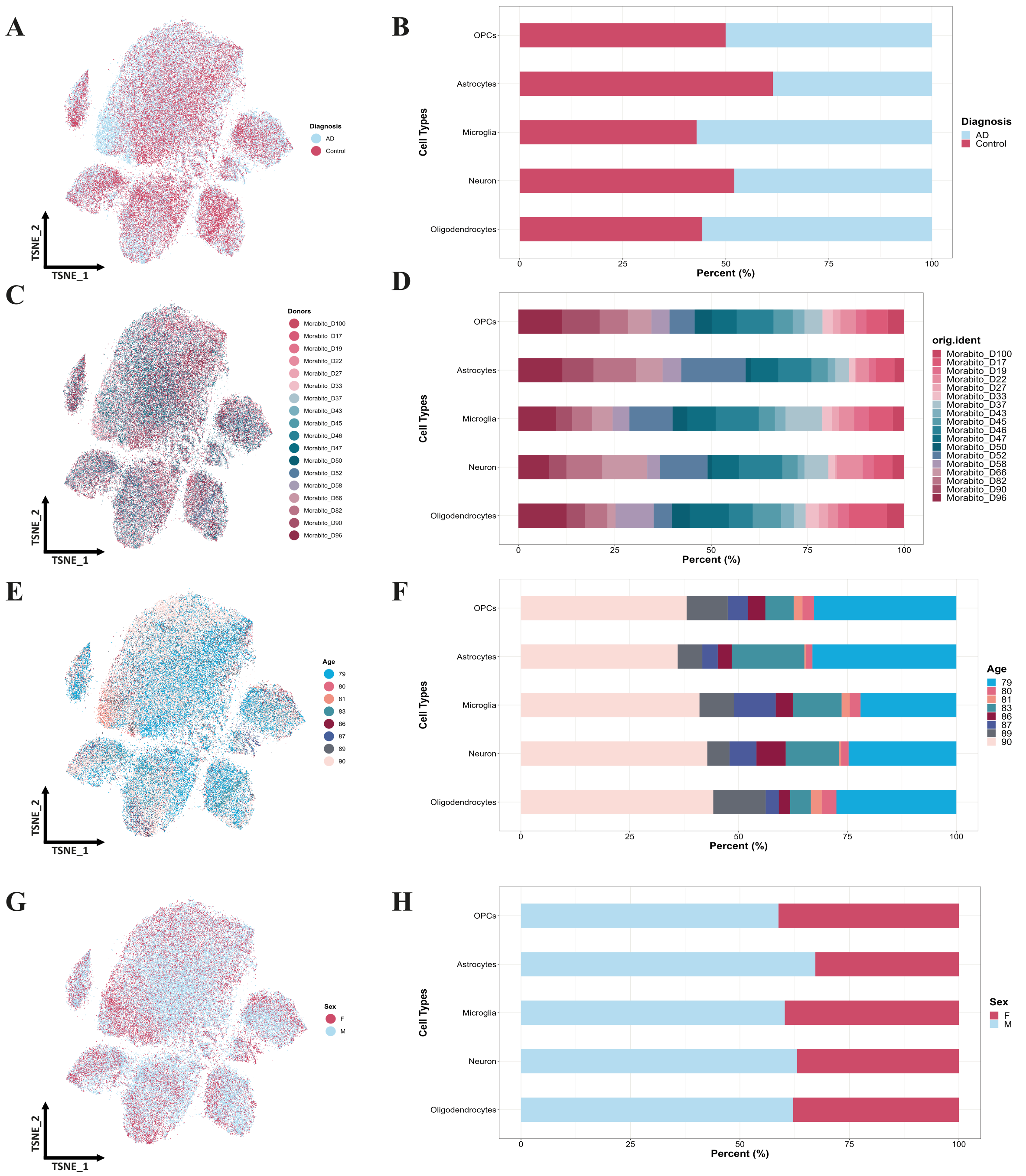

I

Cell Type	Cell Number	AD	Control
Oligodendrocytes	52033	28995	23038
Neuron	24737	11858	12879
Microglia	10973	6263	4710
Astrocytes	9554	3685	5869
OPCs	3840	1922	1918

J

Cluster	Cell Number	AD	Control
0	52033	28995	23038
1	24737	11858	12879
2	10973	6263	4710
3	9554	3685	5869
4	3840	1922	1918

Online Resource-2G. Annotation of Morabito snATAC-seq dataset was validated by checking the distribution of certain features (diagnosis, donor distribution, donor age and sex) across cell types. (A-B) Distribution of cell types based on diagnosis. **(C-D)** Distribution of donors across cell types. **(E-F)** Distribution of donor ages across cell types. **(G-H)** Distributi- on of donor sexes across cell types. **(I-J)** Number of AD and control cells in each cluster and cell type.

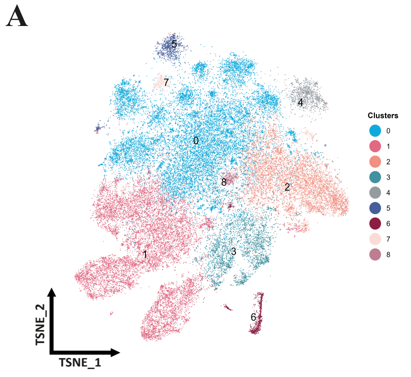

B

Cluster	Cell Number	Morabito	Sadick	AD	Control	Uniquely enriched gene numbers
0	15648	1665	13983	10706	4942	2
1	12295	1294	11001	8301	3994	106
2	6498	470	6028	4633	1865	391
3	3377	238	3139	2515	862	138
4	1275	21	1254	935	340	43
5	943	17	926	673	270	10
6	814	104	710	612	202	425
7	680	3	677	459	221	3
8	490	1	489	319	171	6

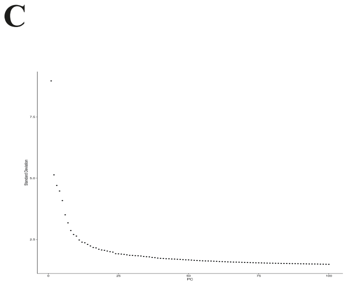

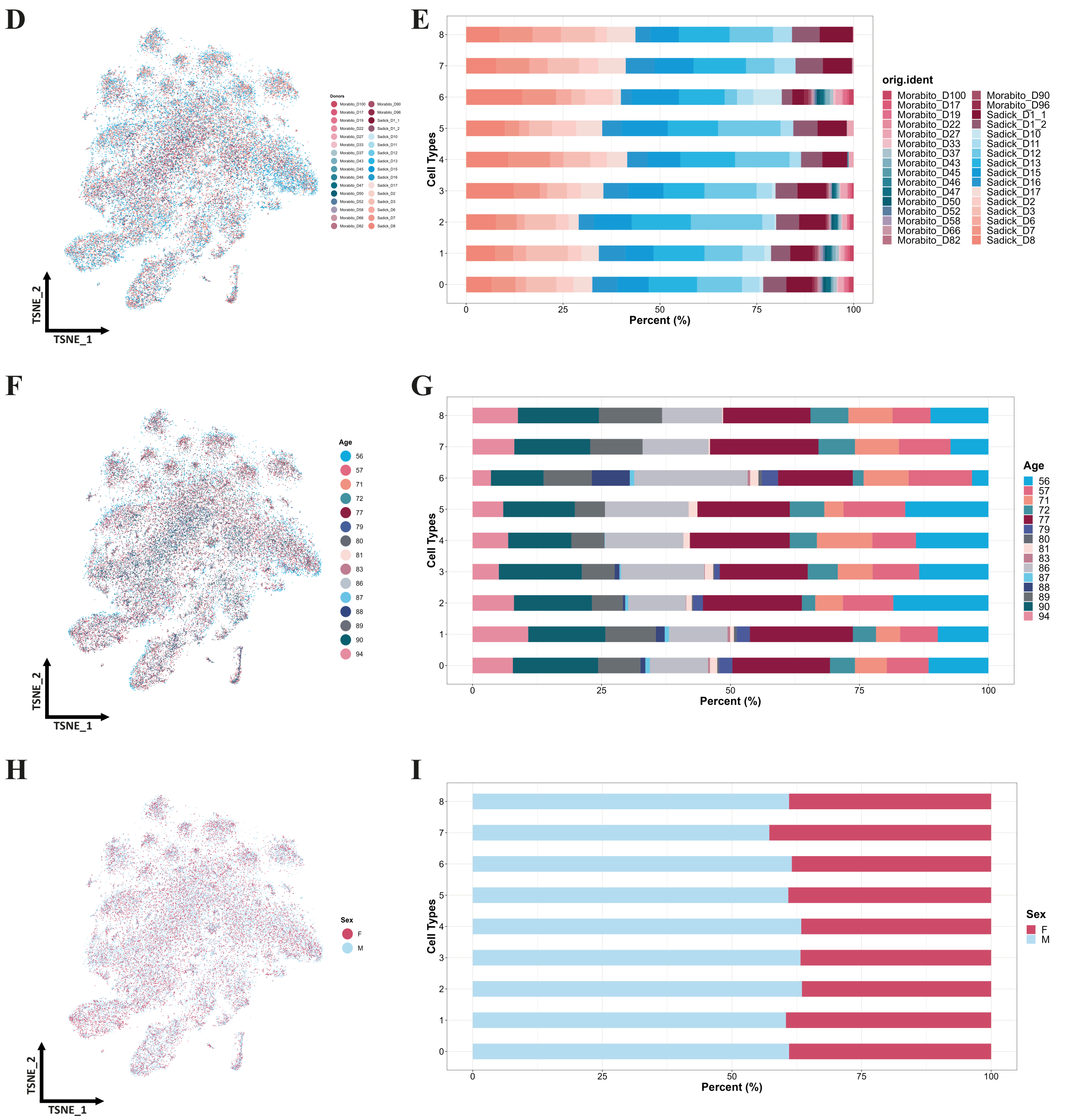

Online Resource-2H. Integration of astrocytes obtained from two datasets was vali- dated by checking the distribution of certain features (diagnosis, donor distribution, donor age and sex) across astrocyte subclusters. (A-C) Astrocye subclusters, number of cells in each cluster, and elbow plot for deciding how many principle components should be used in downstream analyzes. **(D-E)** Distribution of donors across cell types. **(F-G)** Distribution of donor ages across cell types. **(H-I)** Distribution of donor sexes across cell types.

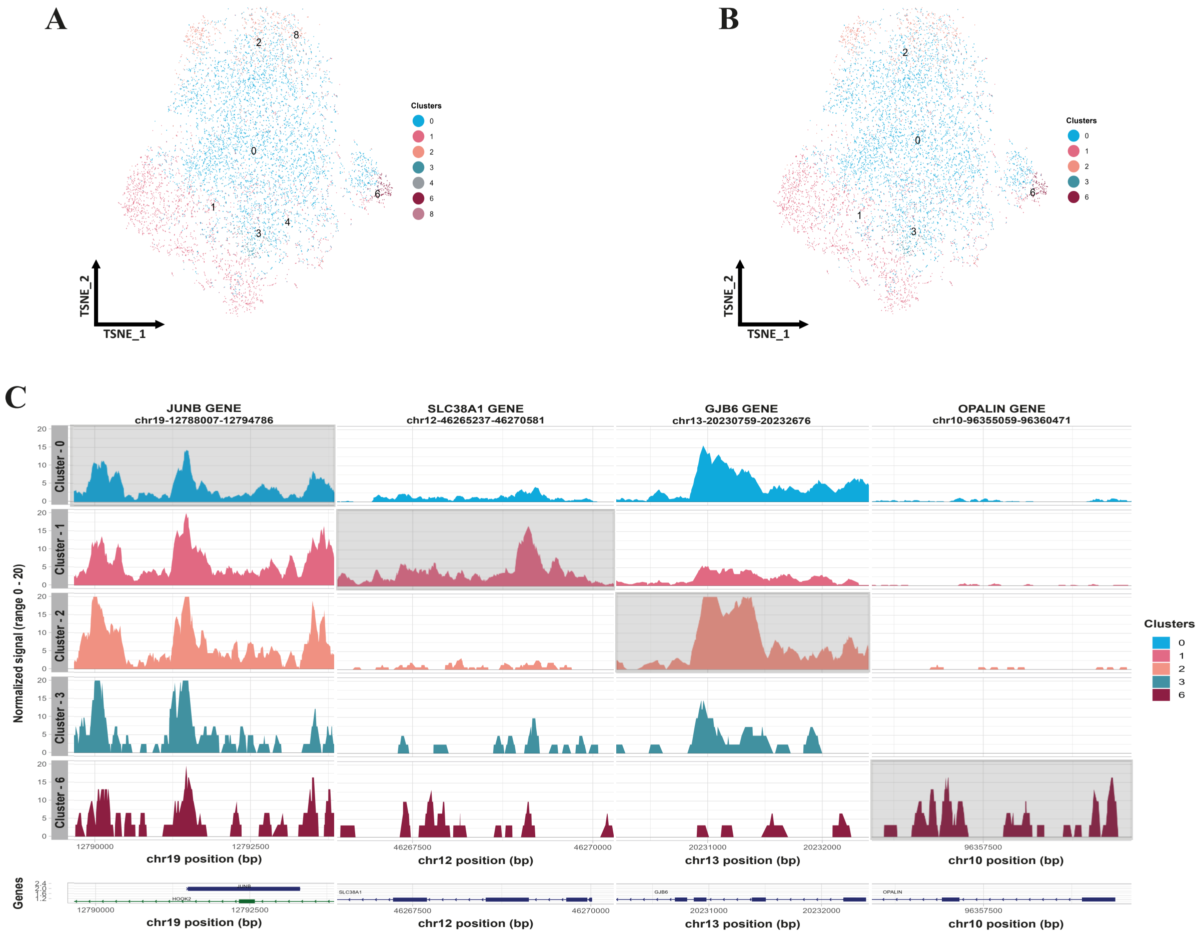

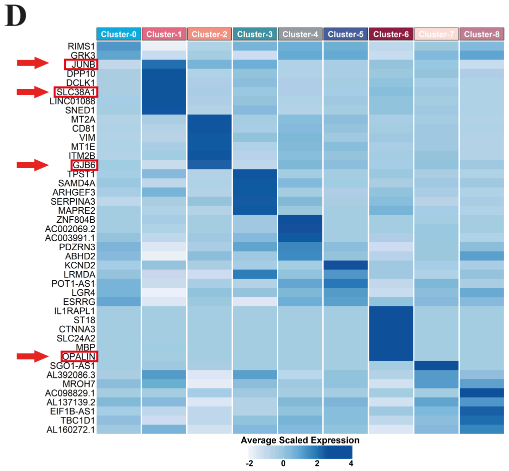

E

Cluster	Mean Prediction Score	Cell Number	AD	Control
Cluster-0	0.448	5632	1826	3806
Cluster-1	0.589	2618	1390	1228
Cluster-2	0.437	954	282	672
Cluster-3	0.408	203	128	75
Cluster-6	0.648	144	59	85
**Overall mean prediction score**	**0.488**			

Online Resource-2I. Multi-modal integration of astrocytes and validation of label transfer process. (A) TSNE plot of astrocytes after label transfer (9554 cells) and after remo- val of cluster-4 and cluster-8 (9551 cells) **(B)** due to low number of cells representing these clusters. **(C)** Promoter accessibility of JUNB, SLC38A1, GJB6 and OPALIN genes across subt- ypes and their gene expression **(D)** in same subtypes. Promoter accessibilty of JUNB gene is decreased in cluster-0 and its gene expression depleted in same cluster. Similarly, while promo- ter accessiblity of SLC38A1, GJB6 and OPALIN genes increased in cluster-1, cluster-2 and cluster-6, expression of those genes were enriched in same respective clusters show the correla- tion between promoter accessibility and gene expression. **(E)** Number of AD and control cells in each cluster and mean prediction scores which shows the accuracy of label transfer process.

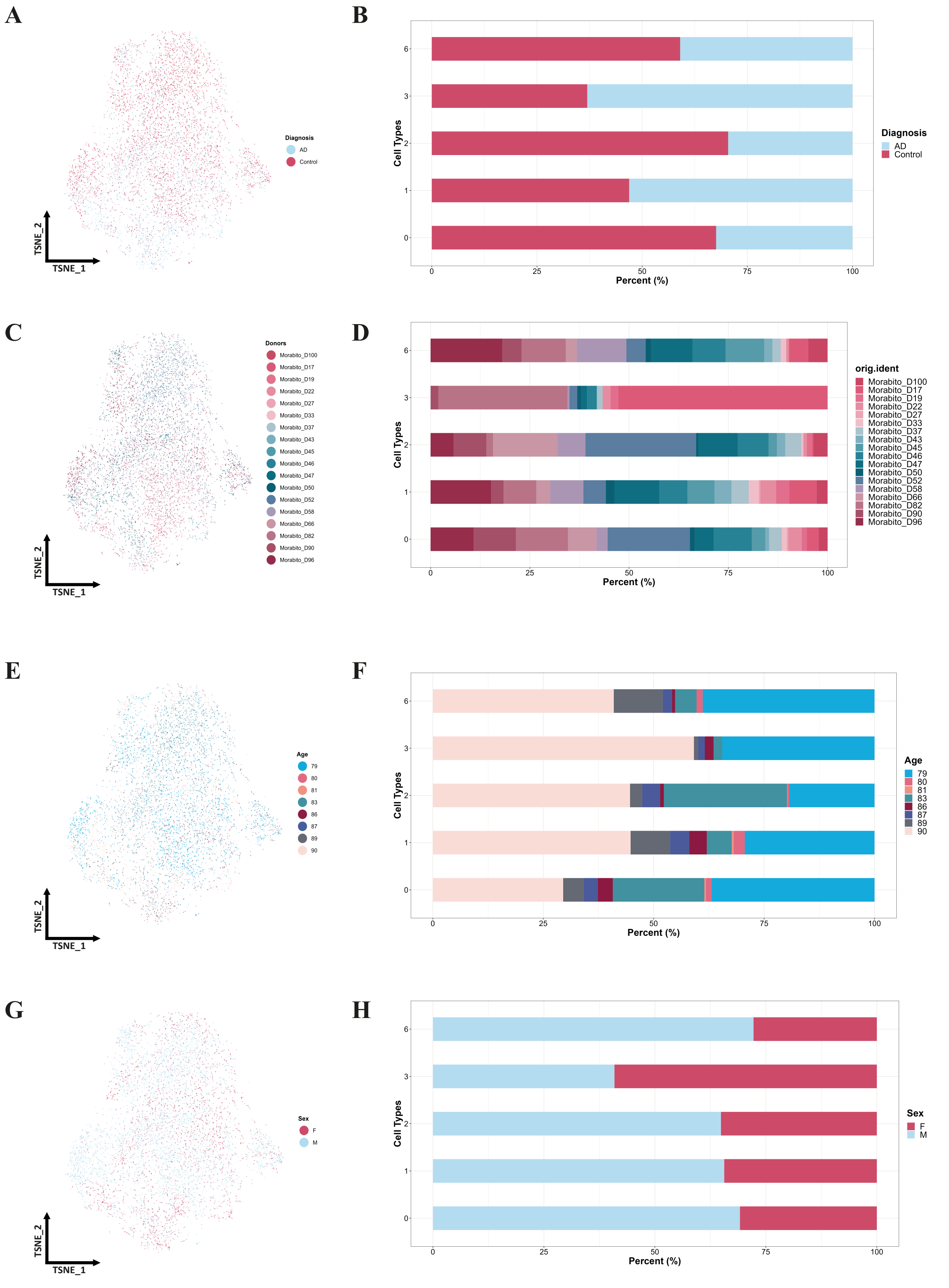

Online Resource-2J. Multi-modal label transfer process was validated by checking the distribution of certain features (diagnosis, donor distribution, donor age and sex) across cell types. (A-B) Distribution of subtypes based on diagnosis. **(C-D)** Distribution of donors across subtypes. **(E-F)** Distribution of donor ages across subtypes. **(G-H)** Distribution of donor sexes across subtypes.

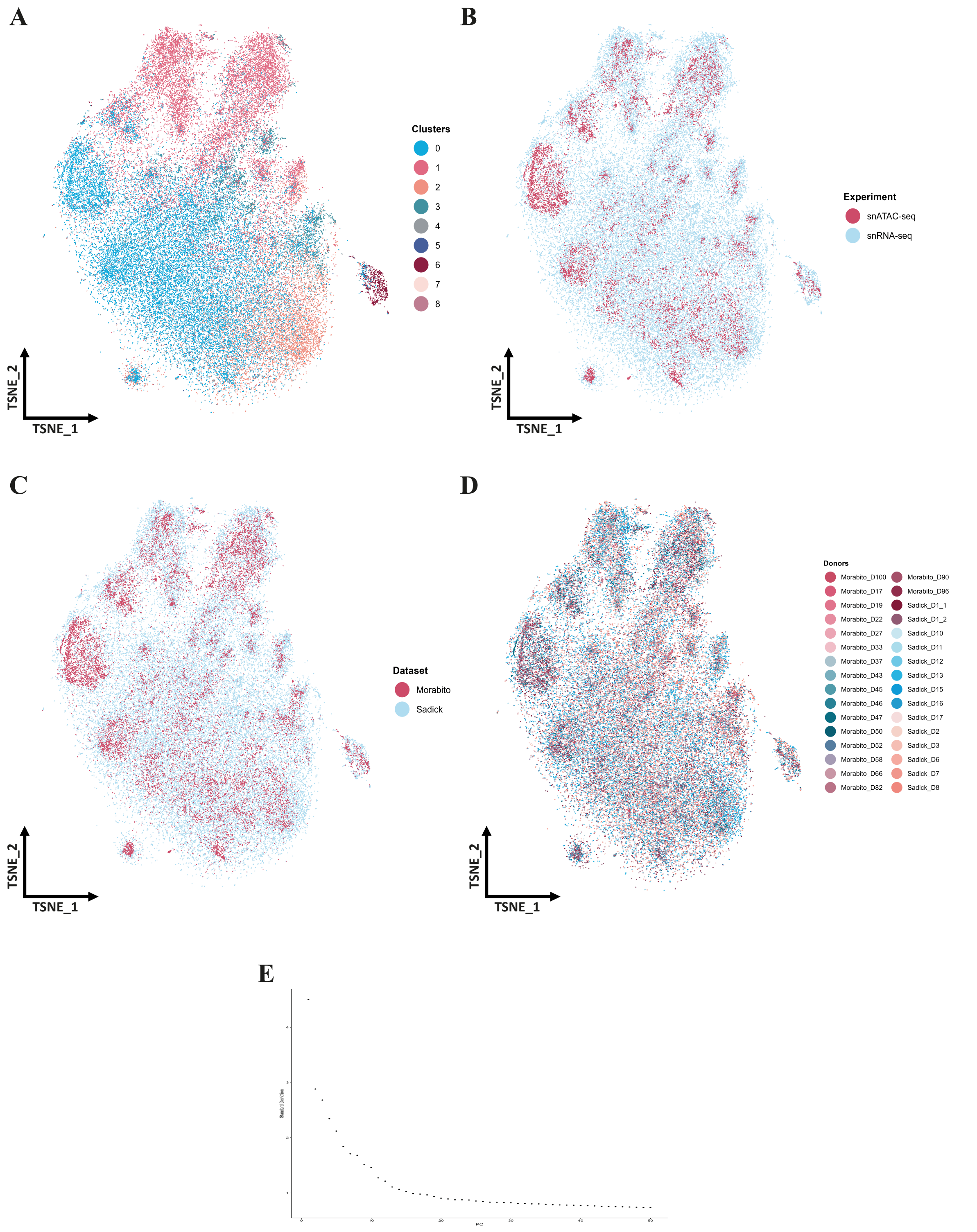

Online Resource-2K. Multi-modal label transfer process was validated by co-embedding snRNA-seq and snATAC-seq astrocytes in a same low-dimensional space and checking the distribution of certain features (experiment, dataset and donor) across subtypes. (A-B) Low dimensional representation of Harmony corrected subtypes from both RNA and ATAC-seq datasets. **(C-D)** Distribution of co-embedded subtypes based on dataset and donors. **(E)** Elbow plot shows how many principle components should be used for co-embedding subtypes from RNA and ATAC-seq data.
