# Supplementary material for: Functional Specificity of Astrocyte Subtypes in Alzheimer’s Disease: Decoding Disease Mechanisms Through Network-based Analysis of Integrated Single-Nuclei Multi-Omic Data
Source: Mol Neurobiol. 2025 Apr 29;62(9):11611–31. doi: 10.1007/s12035-025-04965-8 (PMC12367892; doi:10.1007/s12035-025-04965-8)
Supplement: Supplementary file 1 — Supplementary file1 (ZIP 122312 KB) [file 12035_2025_4965_MOESM1_ESM.zip › ESM_1.pdf]

Article title: Functional Specificity of Astrocyte Subtypes in Alzheimer's Disease: Decoding Disease Mechanisms through Network-based Analysis of Integrated Single-Nuclei Multi-Omic Data

Journal name: Molecular Neurobiology

Author names: Atılay İlgün, Tunahan Çakır

Author affiliations: Gebze Technical University, Department of Bioengineering, Gebze/Kocaeli/TÜRKİYE

Corresponding author: Tunahan Çakır (tcakir@gtu.edu.tr)

snRNA-seq Data Processing for the Sadick Dataset

Gene expression count matrices from the donors in the Sadick dataset [1] (5 control, 9 AD) were introduced into R independently. Cells in each donor were filtered based on the number of unique transcripts per cell, the total number of transcripts per cell and percentage of mitochondrial genes per cell as recommended by Seurat (see Online Resource 1A-E for the distribution of unfiltered & filtered quality control metrics for the Sadick dataset). After filtering, the raw count matrix of each donor was independently normalized with the sctransform::SCTransform (v0.3.5) [2] function. This function does not only normalize the count data, but it also scales the data and identifies variable features. For each donor, 2000 variable genes were identified, and the percentages of transcripts corresponding to mitochondrial, ribosomal, and heat-shock proteins as well as hemoglobin transcripts were regressed out during this normalization step. Afterwards, Seurat::SelectIntegrationFeatures function was used to select a new set of 2000 features from the donor-derived variable features, to be used in integration. Then, transcripts for mitochondrial, ribosomal, and heat-shock proteins and hemoglobin were removed among those variable features, leaving 1989 variable features. In the next step, the normalized data was prepared for Canonical Correlation Analysis (CCA) [3] by Seurat::PrepSCTIntegration function. Since, the batch differences across samples were significant in this dataset, we implemented integration by CCA by using Seurat::FindIntegrationAnchors and Seurat::IntegrateData functions respectively (dims=50). Seurat::FindIntegrationAnchors function was used to identify anchor cells across different donors. Those anchor cells are pair of cells from each donor, and they were used as input for Seurat::IntegrateData function. After the integration, Principle Component Analysis (PCA) was implemented for linear dimension reduction by using Seurat::RunPCA function, and it was followed by non-linear dimension reduction method t-Distributed Stochastic Neighbor Embedding (tSNE) by using first 50 principle components (PC). Non-linear dimension reduction was implemented by Seurat::RunTSNE function and followed by the computation of Shared Nearest-Neighbor (SNN) by using Seurat::FindNeighbors function (dims=50). Subsequently, cell clustering was performed by the Louvain algorithm by using Seurat::FindClusters function (0.15 resolution). Then, non-parametric Wilcoxon Rank Sum Test was applied on the raw counts across clusters by using Seurat::FindAllMarkers function to determine differentially expressed cell type markers in each cluster. These differentially expressed (Bonferroni correction was implemented, fold change > 1.20) cell markers were used to annotate the identified clusters (see Online Resource 3 for differential testing results and see Online Resource 2B and 2C for details of cell type annotation). Since cluster-4 was predominantly composed of cells from AD donors (Online Resource 2A), it was removed prior to cluster annotation. After the annotation of major CNS cell types, astrocytes were subsetted and saved for downstream integration processes.

snRNA-seq Data Processing for the Morabito Dataset

Gene expression count matrices from the donors of the Morabito dataset [4] (7 control, 11 AD) were introduced into R. The same quality control metrics as mentioned for the Sadick dataset were assessed, and filtrations, normalizations and feature selections were performed, leading to 1995 variable genes (see Online Resource 1F-I for the distribution of unfiltered & filtered quality control metrics for the Morabito dataset). Different from the analysis of Sadick snRNA-seq data analysis, no integration or batch correction was implemented here. After merging the donors, PCA was implemented and it was followed by Seurat::ElbowPlot function to determine the number of principle components to be used in downstream analyses. tSNE, SNN, the Louvain algorithm and cluster annotation steps were applied as mentioned in the previous section for the Sadick dataset (see Online Resource 3 for differential testing results), and astrocytes were subsetted and saved for downstream integration processes after the annotation of major CNS cell types (Online Resource 2D-E).

Co-Expression Significance in Cluster-3

We used the ggpubr::stat_cor function in R to test the co-expression significance in cluster-3. Spearman correlation coefficients and p-values were given as outputs by the function. The function displays the results of cor.test() function. Within this function, p-values are calculated by the usage of AS 89 algorithm for n < 1290 and exact = TRUE, otherwise with the asymptotic t approximation.

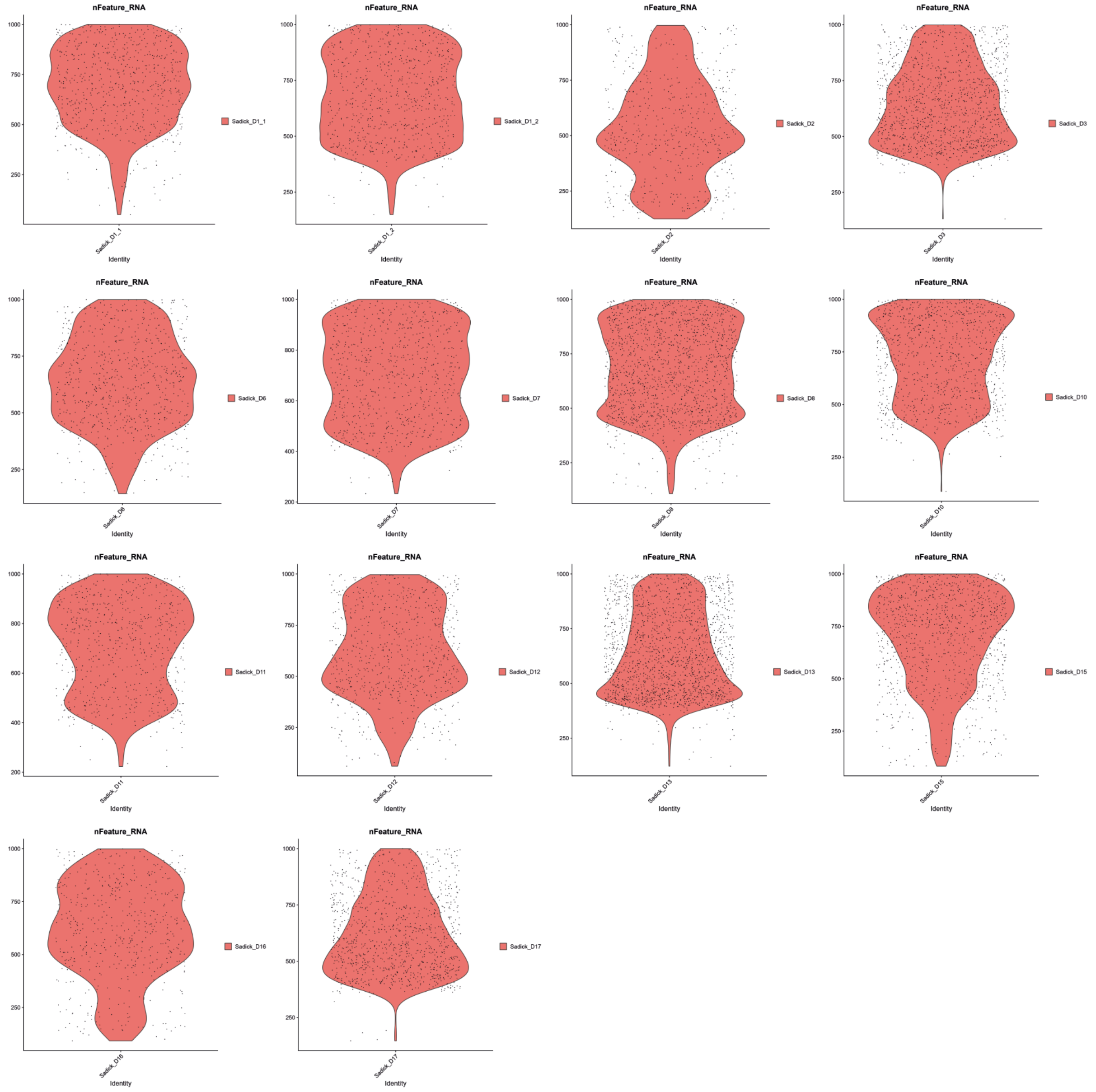

Online Resource-1A. Quality control assessment of unique number of genes detected per cell (nFeature_RNA) metric (lower bound) in Sadick snRNA-seq dataset. Violin plots show the distribution in the number of unique genes detected per cell across each donor in the Sadick dataset. To ensure appropriate data filtering from the lower end, the y-axis was constrained.

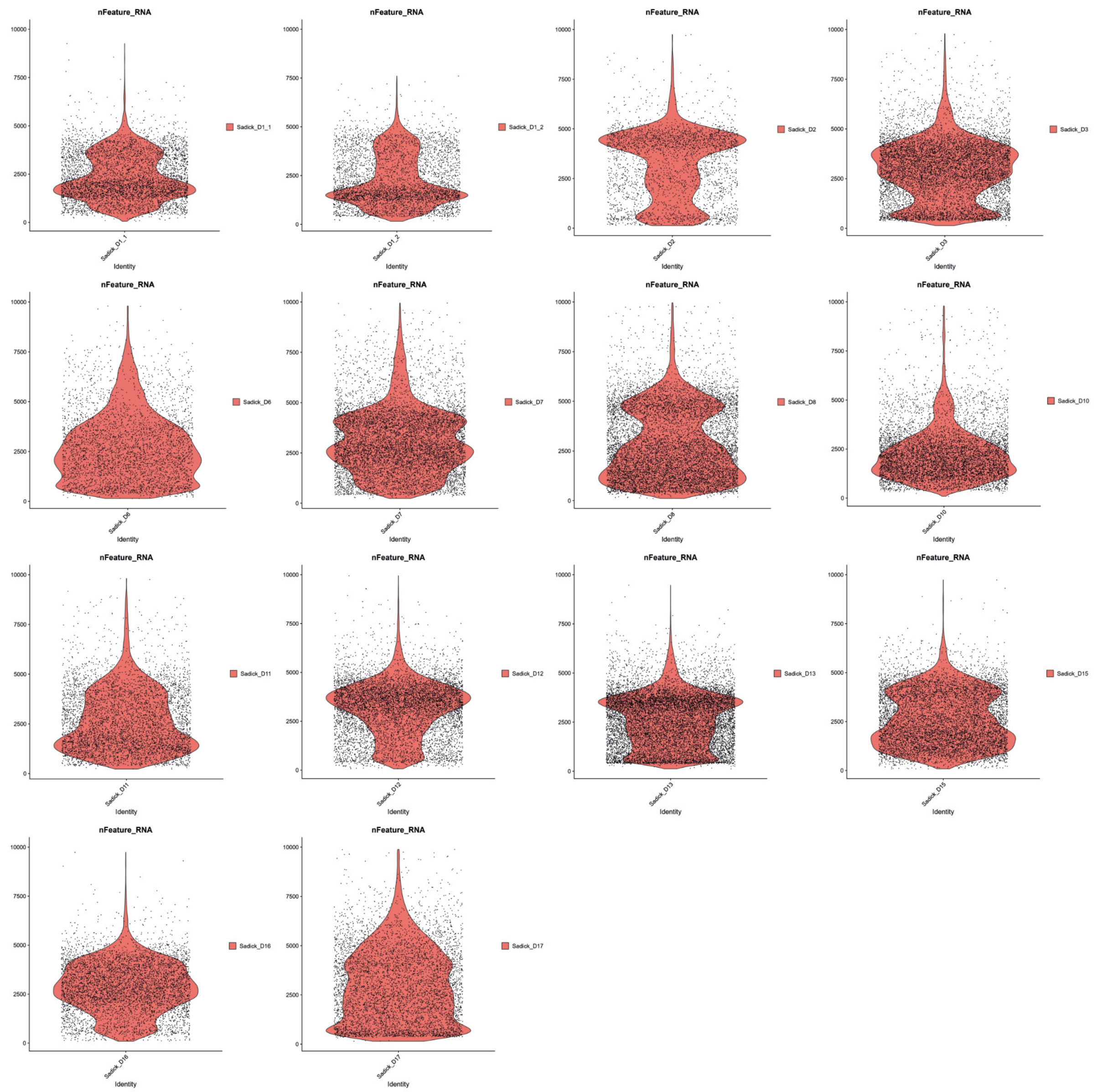

Online Resource-1B. Quality control assessment of unique number of genes detected per cell (nFeature_RNA) metric (upper bound) in Sadick snRNA-seq dataset. Violin plots show the distribution in the number of unique genes detected per cell across each donor in the Sadick dataset. To ensure appropriate data filtering from the upper end, the y-axis was constrained.

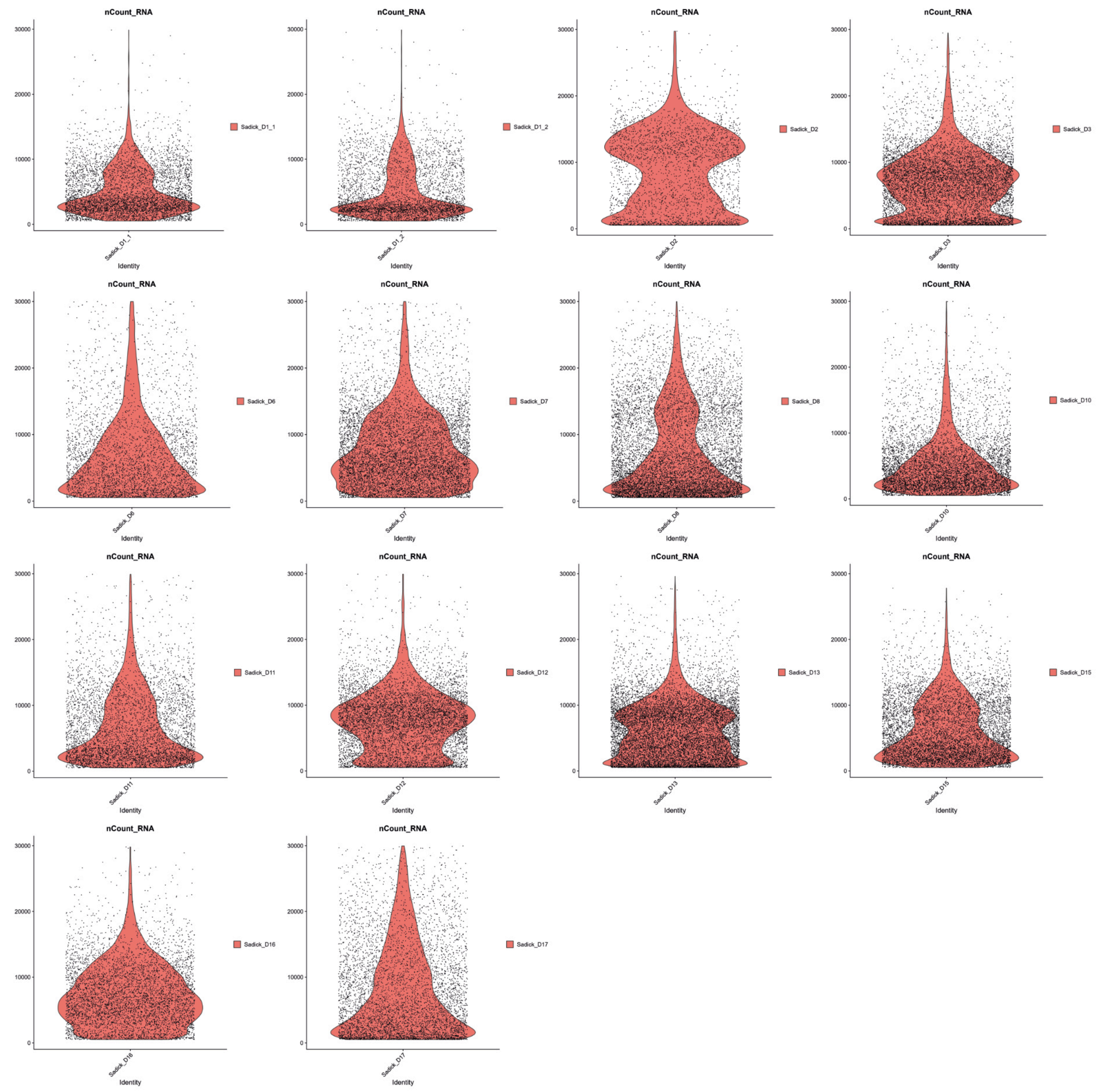

Online Resource-1C. Quality control assessment of total number of gene counts detected per cell (nCount_RNA) metric (upper bound) in Sadick snRNA-seq dataset. Violin plots show the distribution in the total number of molecules detected per cell across each donor in the Sadick dataset. To ensure appropriate data filtering from the upper end, the y-axis was constrained.

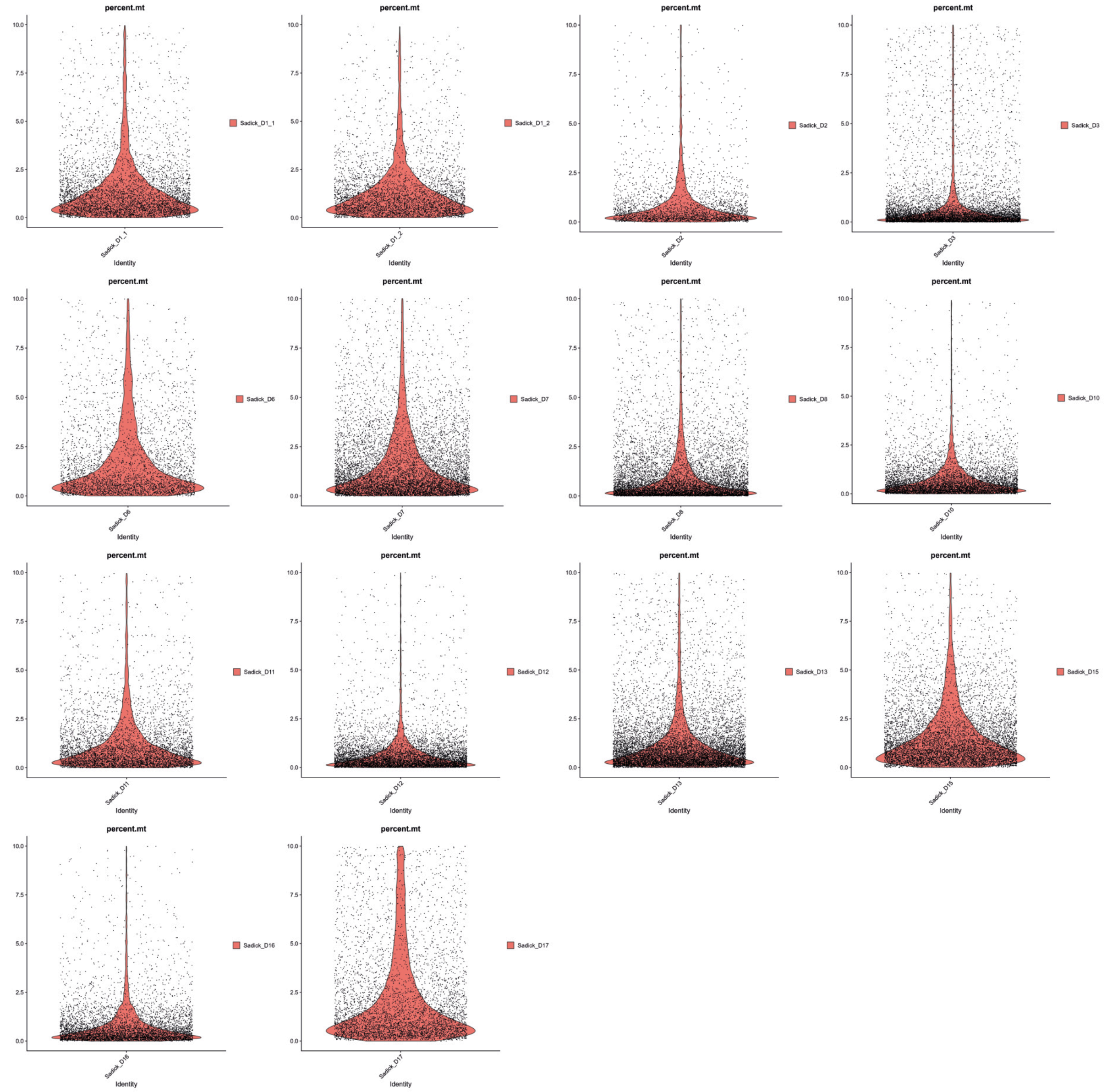

Online Resource-1D. Quality control assessment of percentages of mitochondrial genes per cell (percent.mt) metric in Sadick snRNA-seq dataset. Violin plots show the distribution in the percentages of mitochondrial genes detected per cell across each donor in the Sadick dataset. Cells with high percentage of mitochondrial genes were excluded from the analysis because they could potentially be dying cells.

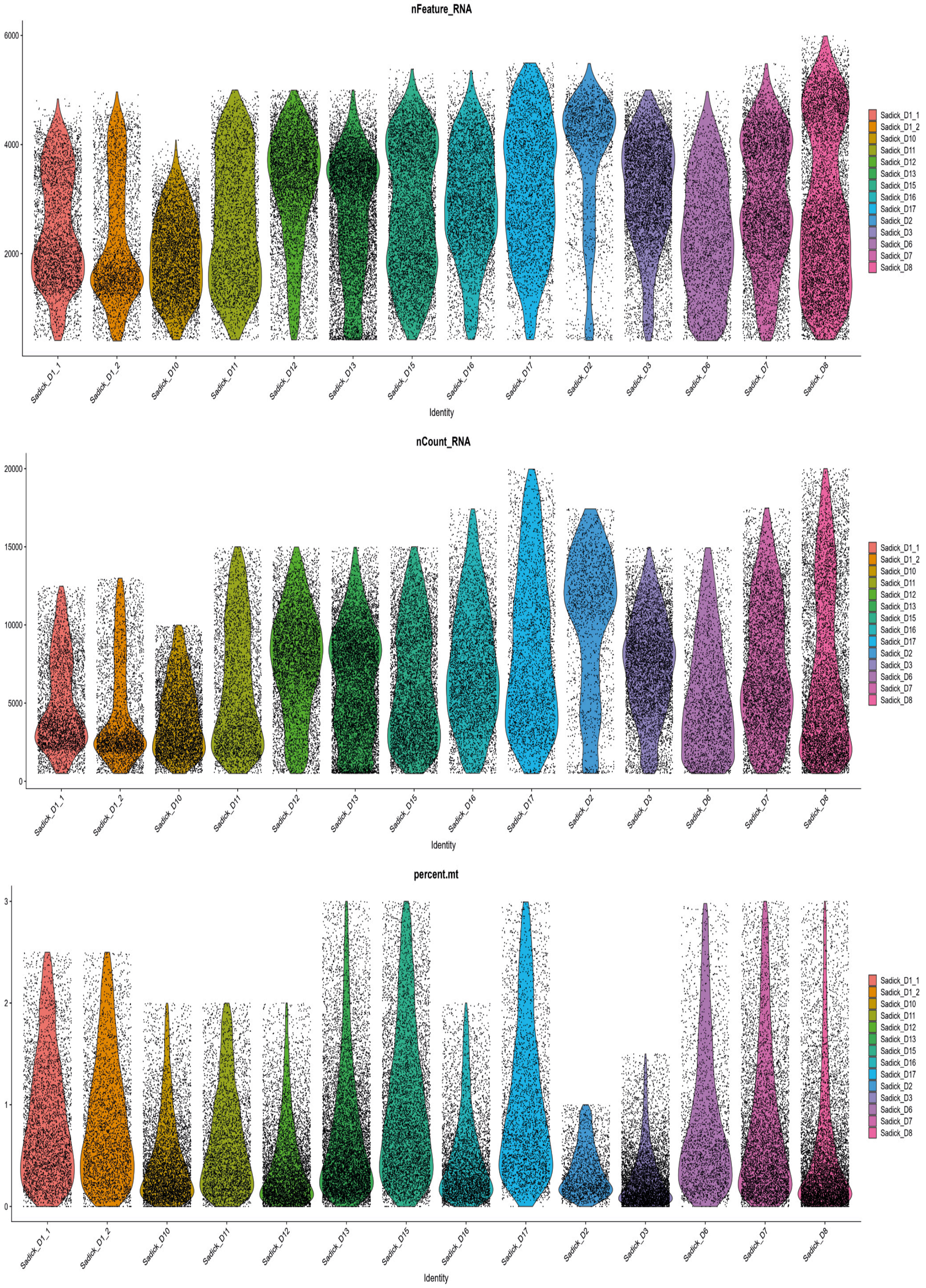

Online Resource-1E. Assesment of all quality control metrics in Sadick snRNA-seq dataset after filtration. Violin plots show the distribution of quality control metrics “nFeature_RNA”, “nCount_RNA” and “percent.mt” across donors of Sadick dataset after filtration.

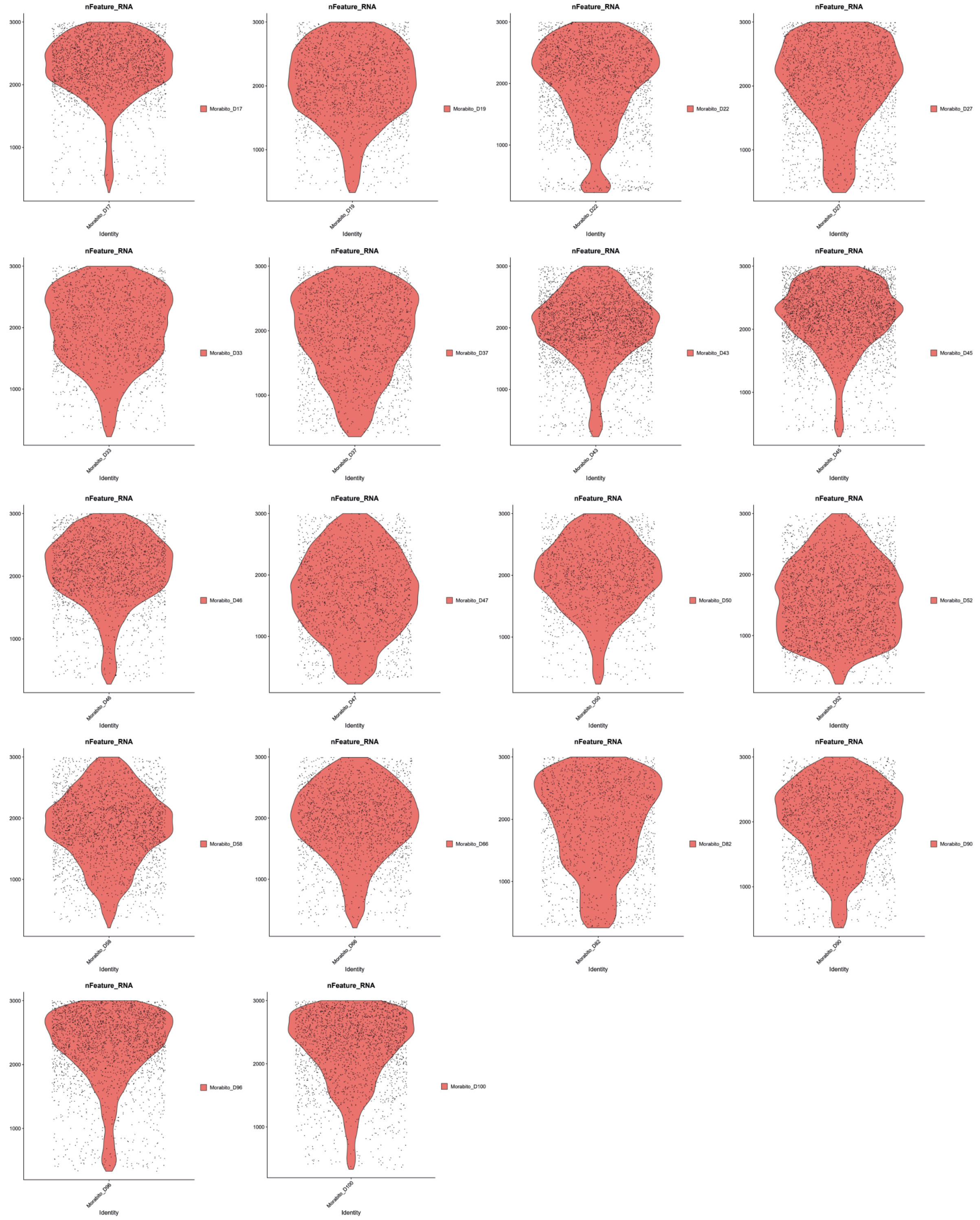

Online Resource-1F. Quality control assessment of unique number of genes detected per cell (nFeature_RNA) metric (lower bound) in Morabito snRNA-seq dataset. Violin plots show the distribution in the number of unique genes detected per cell across each donor in the Morabito dataset. To ensure appropriate data filtering from the lower end, the y-axis was constrained.

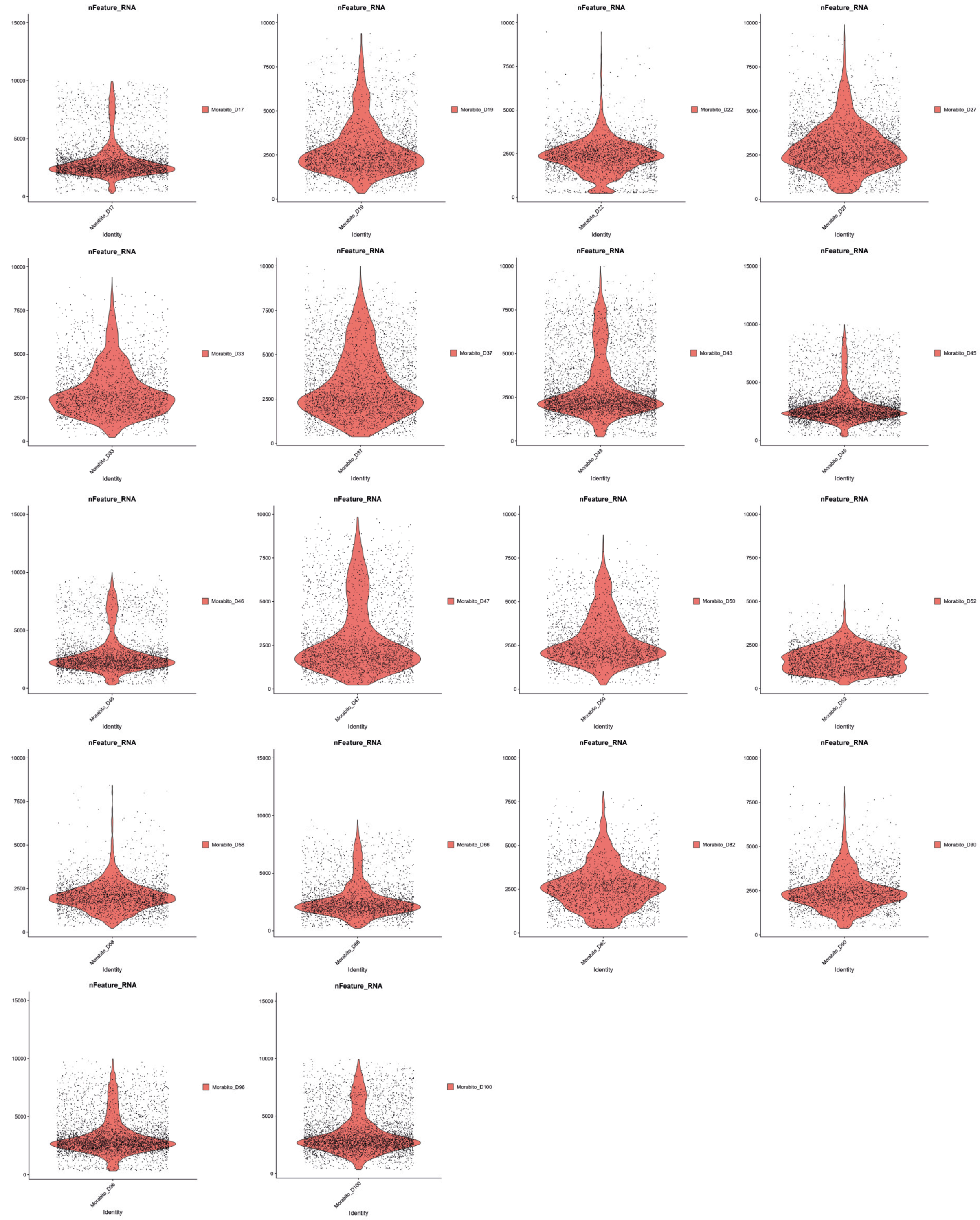

Online Resource-1G. Quality control assessment of unique number of genes detected per cell (nFeature_RNA) metric (upper bound) in Morabito snRNA-seq dataset. Violin plots show the distribution in the number of unique genes detected per cell across each donor in the Morabito dataset. To ensure appropriate data filtering from the upper end, the y-axis was constrained.

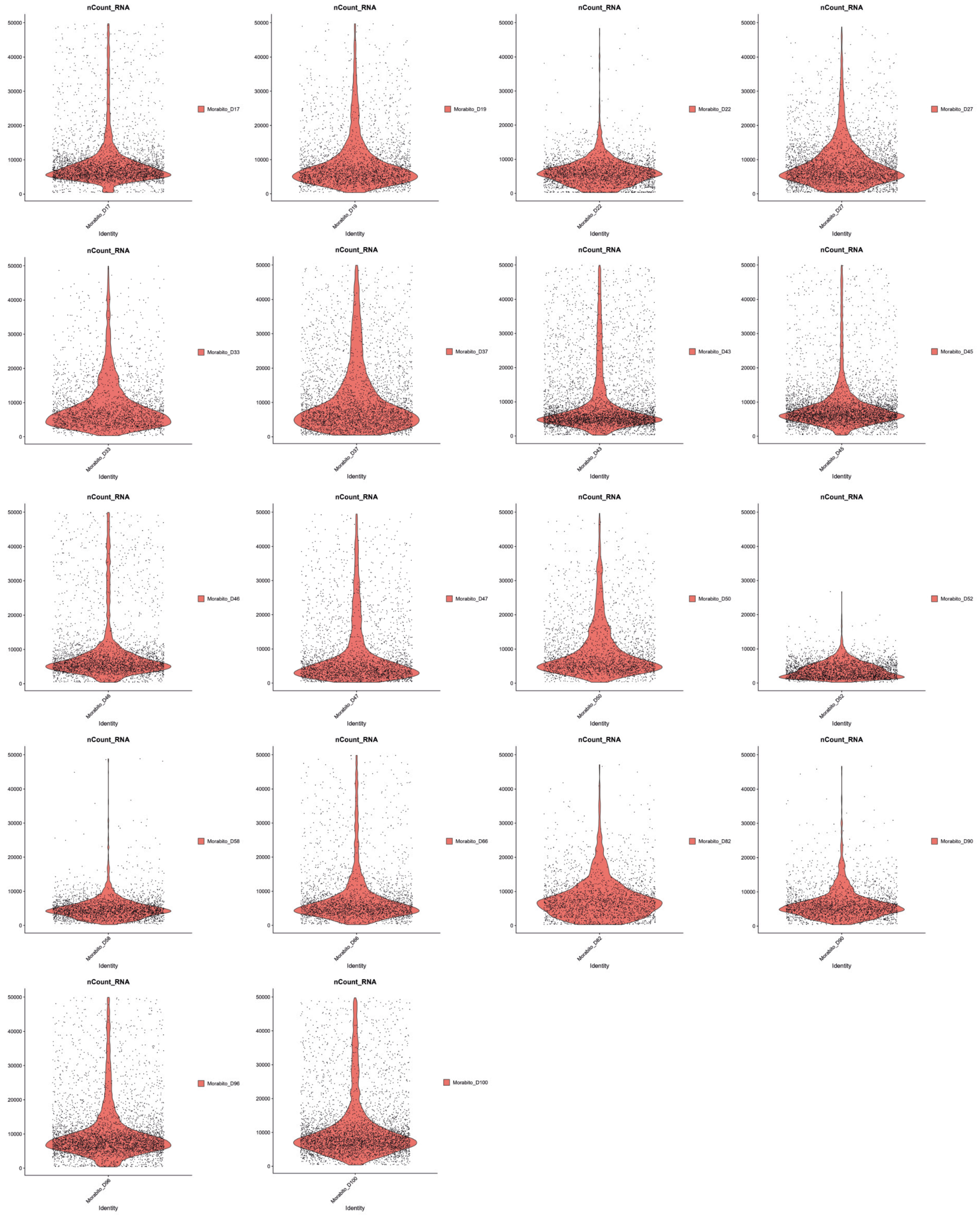

Online Resource-1H. Quality control assessment of total number of gene counts detected per cell (nCount_RNA) metric (upper bound) in Morabito snRNA-seq dataset. Violin plots show the distribution in the total number of molecules detected per cell across each donor in the Sadick dataset. To ensure appropriate data filtering from the upper end, the y-axis was constrained.

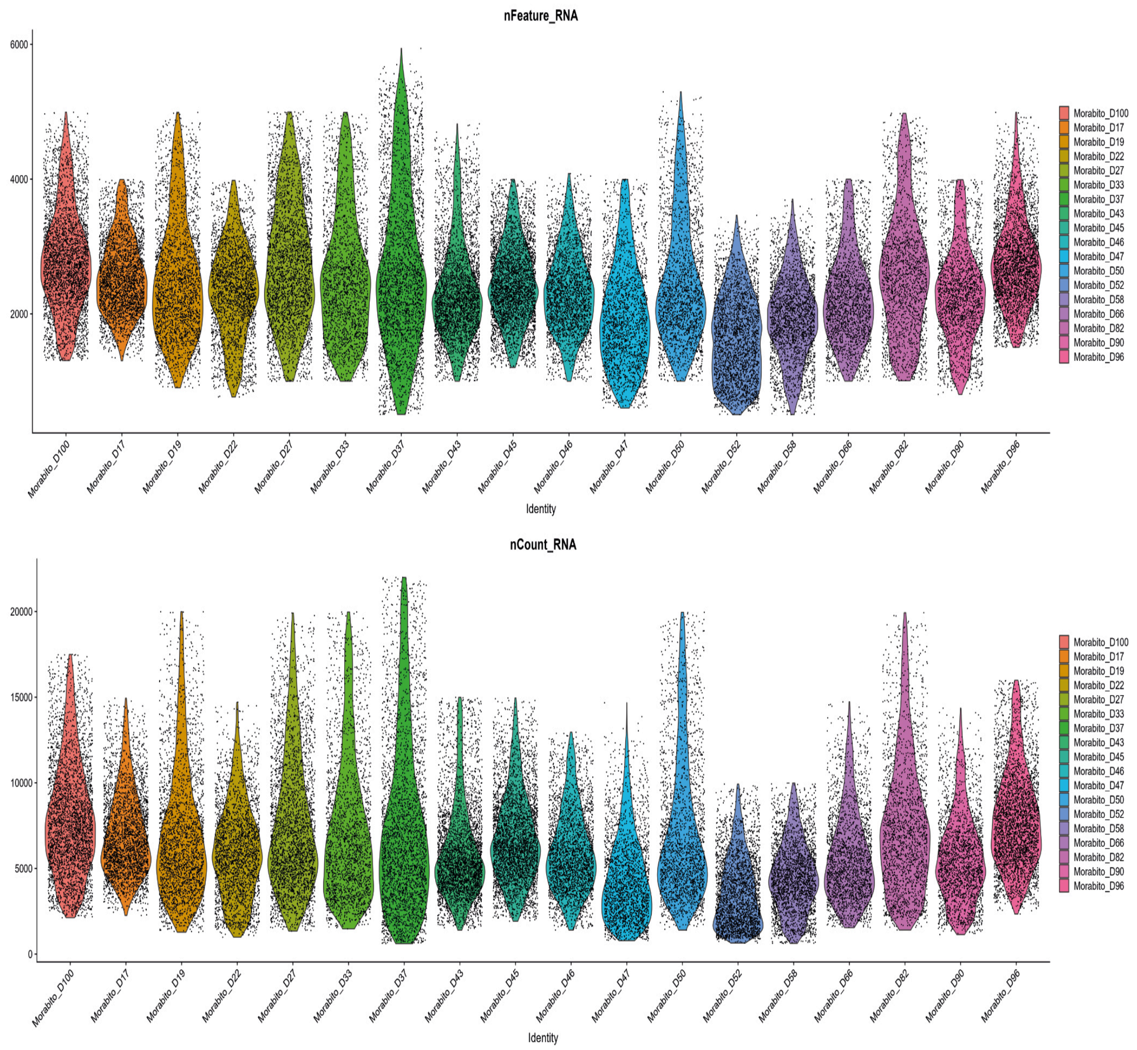

Online Resource-1I. Assesment of all quality control metrics in Morabito snRNA-seq dataset after filtration. Violin plots show the distribution of quality control metrics (nFeature_RNA) and "nCount_RNA" across donors of Morabito dataset after filtration.

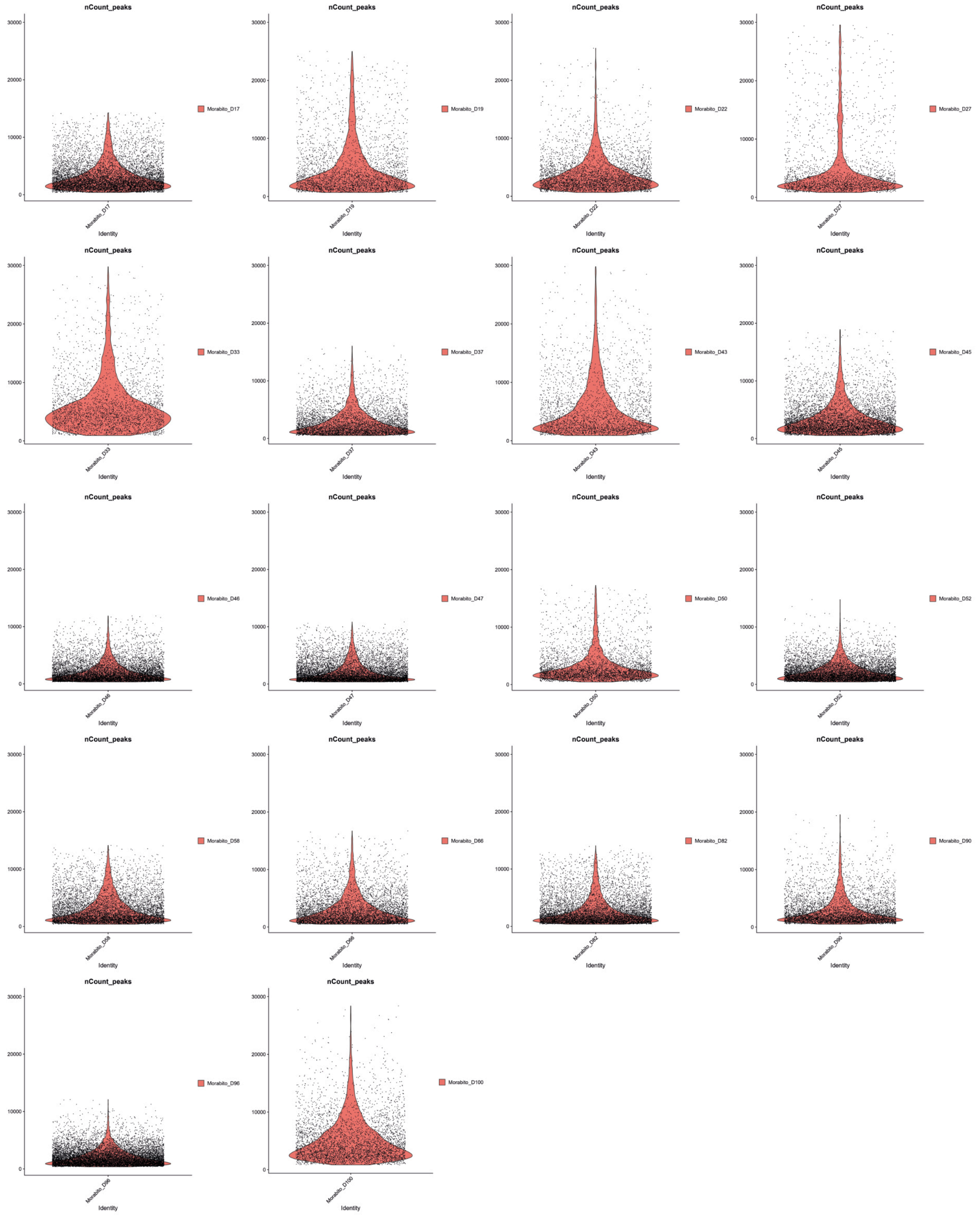

Online Resource-1J. Quality control assessment of total number of peaks detected per cell (nCount_peaks) metric (upper bound) in Morabito snATAC-seq dataset. Violin plots show the distribution in the total number of peaks detected per cell across each donor in the Morabito snATAC-seq dataset. To ensure appropriate data filtering from the upper end, the y-axis was constrained.

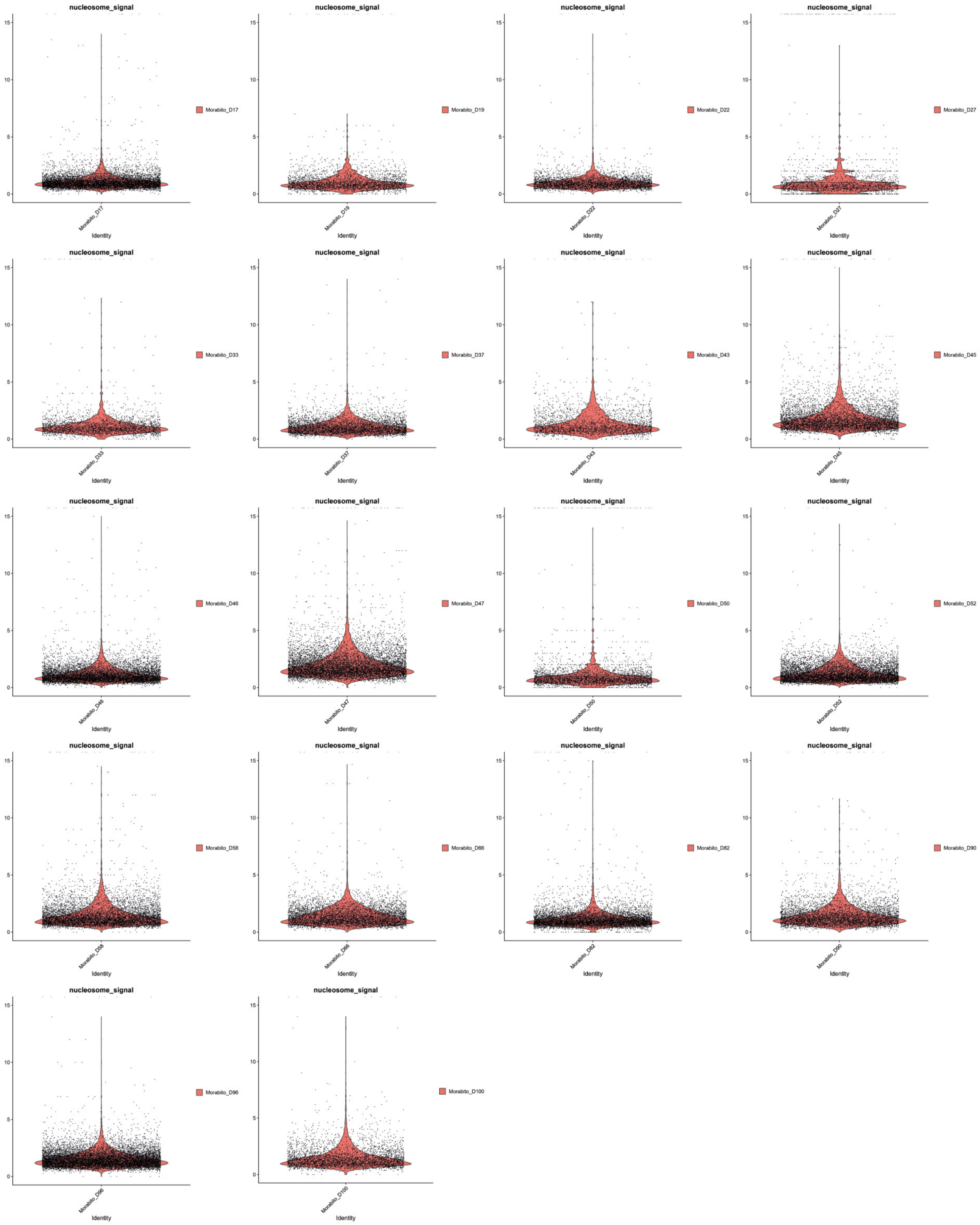

Online Resource-1K. Quality control assessment of nucleosomal signal detected per cell (nucleosome_signal) metric (upper bound) in Morabito snATAC-seq dataset. Violin plots show the distribution of nucleosomal signal per cell across each donor in the Morabito snATAC-seq dataset. To ensure appropriate data filtering from the upper end, the y-axis was constrained.

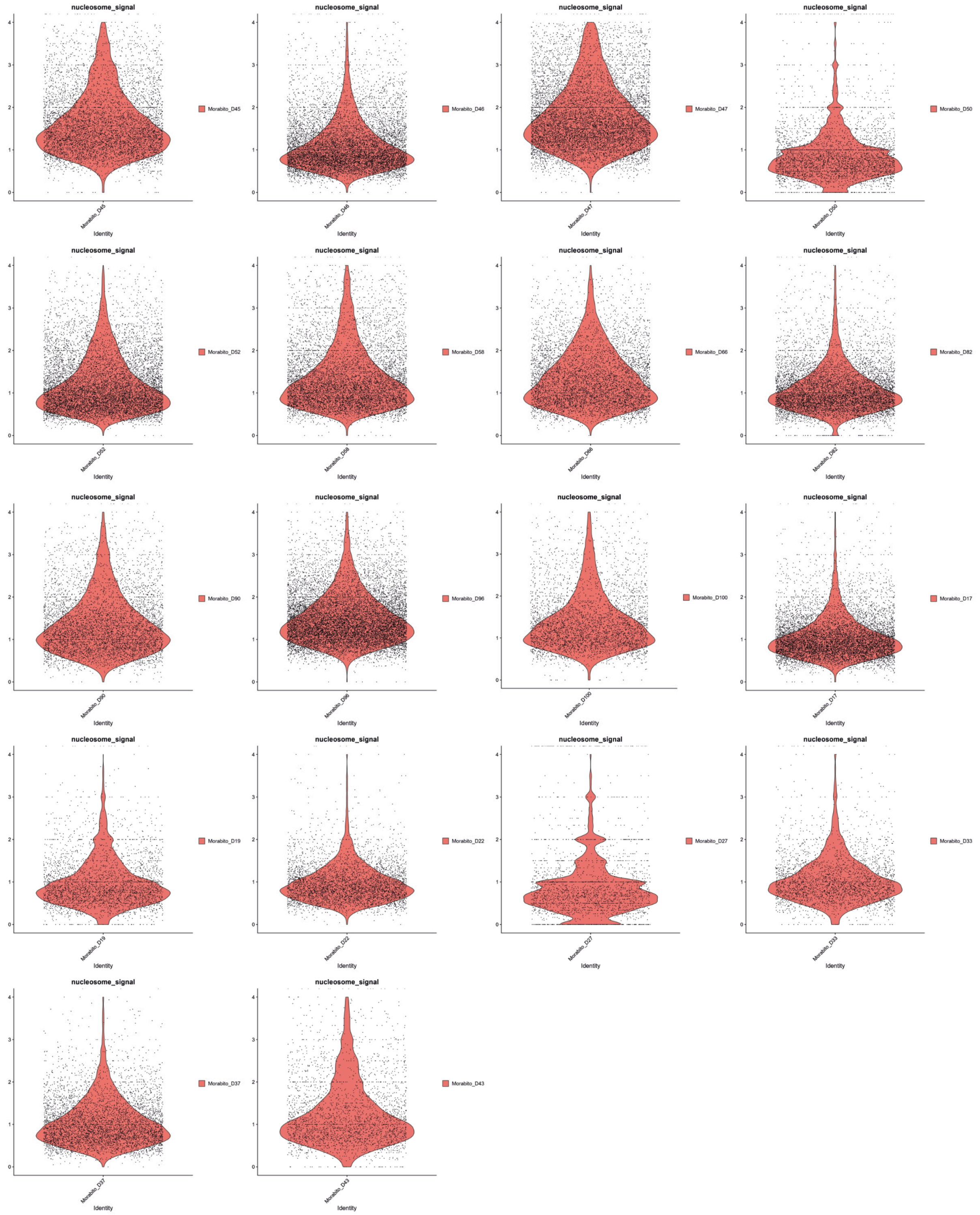

Online Resource-1L. Quality control assessment of nucleosomal signal detected per cell (nucleosome_signal) metric (lower bound) in Morabito snATAC-seq dataset. Violin plots show the distribution of nucleosomal signal per cell across each donor in the Morabito snATAC-seq dataset. To ensure appropriate data filtering from the lower end, the y-axis was constrained.

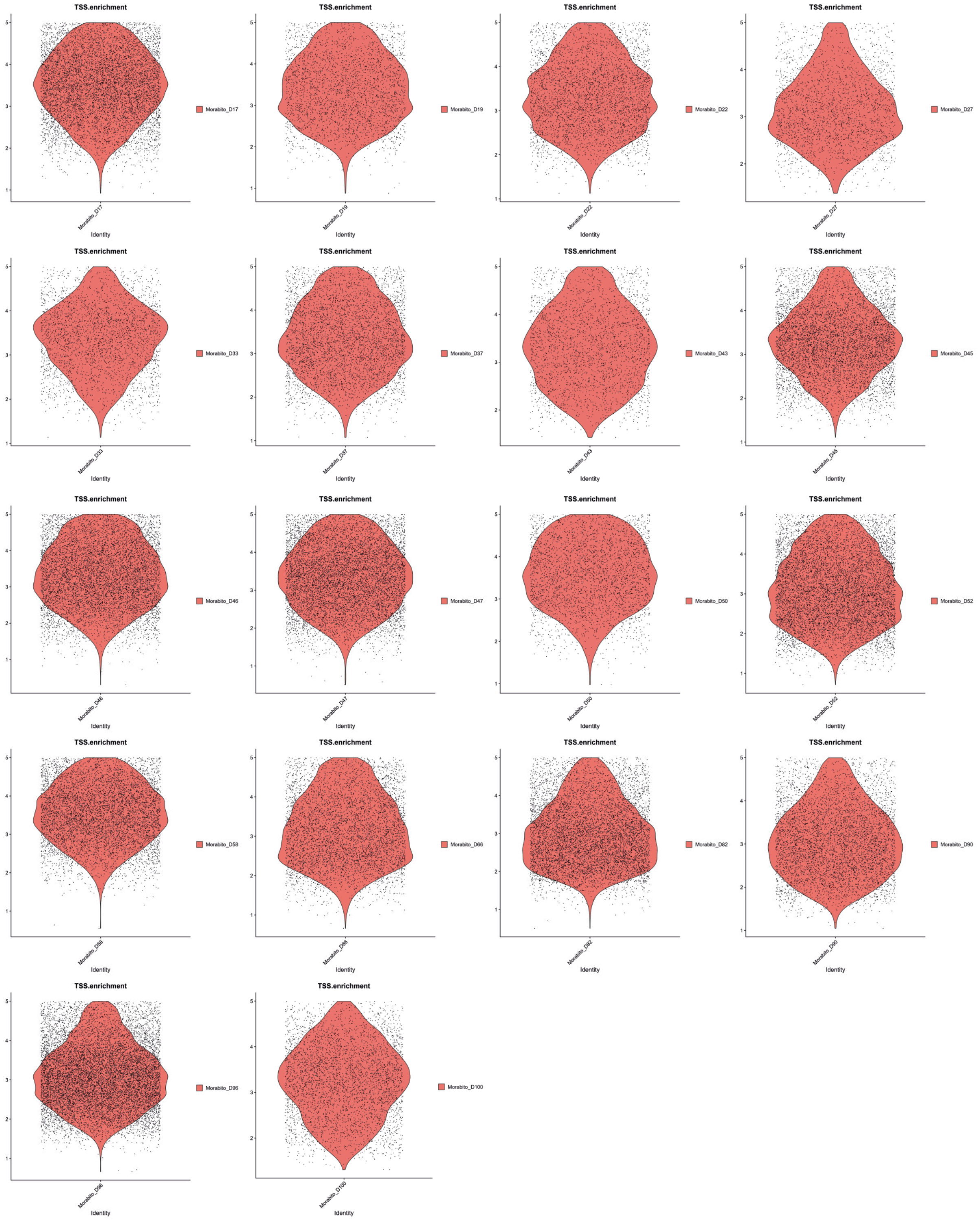

Online Resource-1M. Quality control assessment of TSS enrichment score (TSS.enrichment) metric (lower bound) in Morabito snATAC-seq dataset. Violin plots show the distribution of Transcriptional start site (TSS) enrichment score across each donor in the Morabito snATAC-seq dataset. To ensure appropriate data filtering from the lower end, the y-axis was constrained.

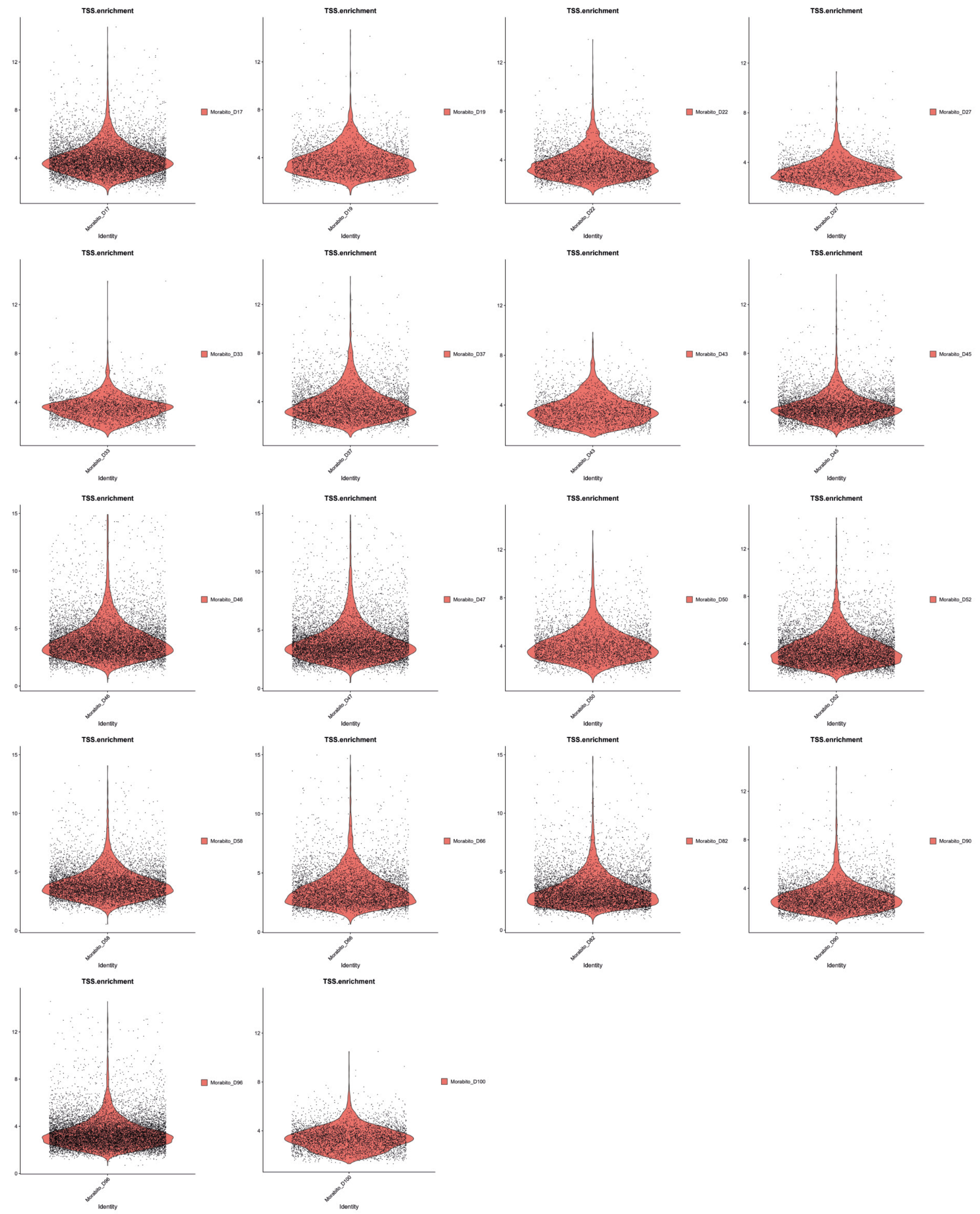

Online Resource-1N. Quality control assessment of TSS enrichment score (TSS.enrichment) metric (upper bound) in Morabito snATAC-seq dataset. Violin plots show the distribution of Transcriptional start site (TSS) enrichment score across each donor in the Morabito snATAC-seq dataset. To ensure appropriate data filtering from the lower end, the y-axis was constrained.

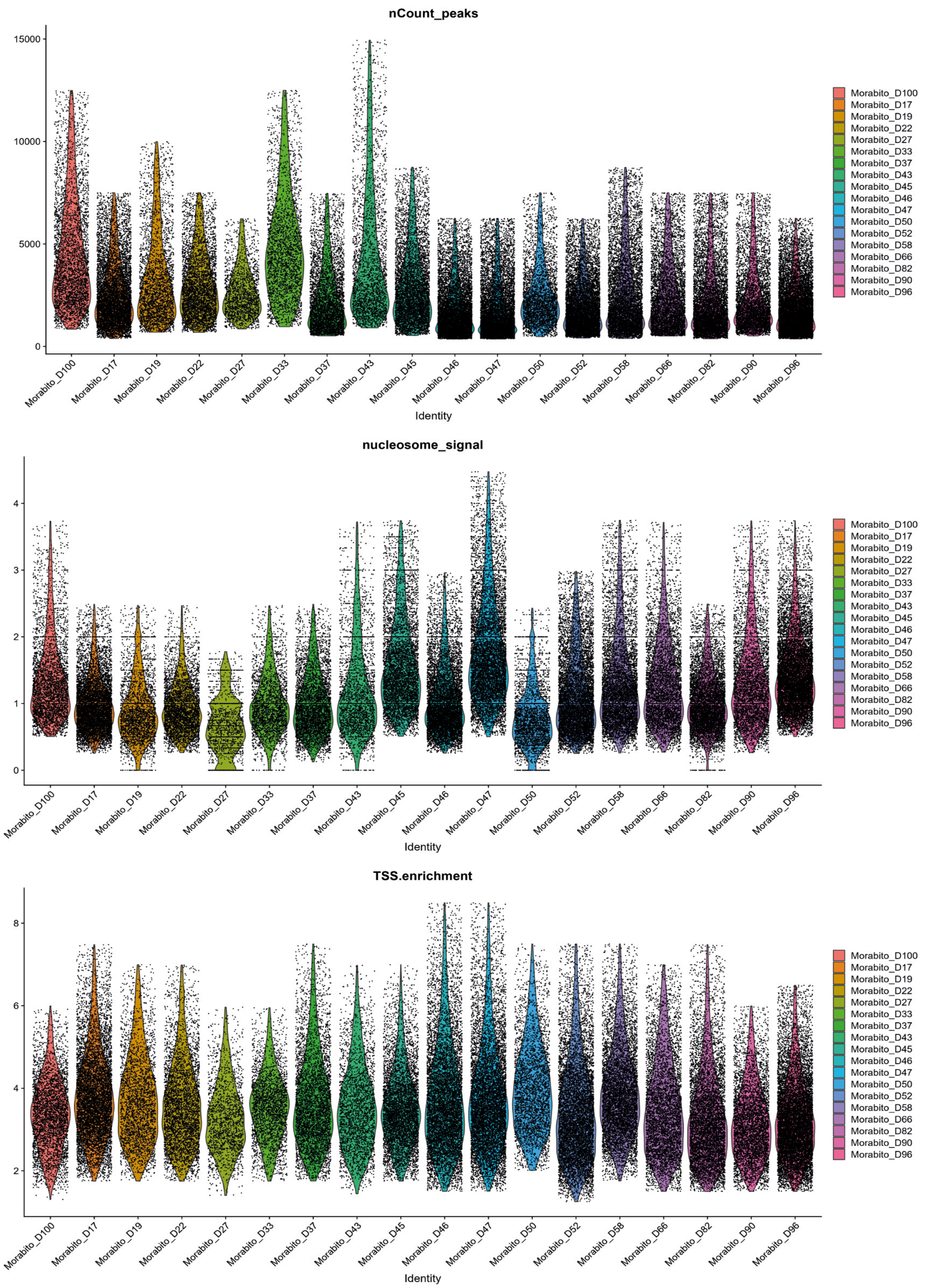

Online Resource-1O. Assesment of all quality control metrics in Morabito snATAC-seq dataset after filtration. Violin plots show the distribution of quality control metrics “nCount_peaks”, “nucleosomal_signal” and “TSS.enrichment” across donors of Morabito snATAC-seq dataset after filtration.

REFERENCES

1. Sadick JS, O'Dea MR, Hasel P, Dykstra T, Faustin A, Liddelow SA (2022) Astrocytes and oligodendrocytes undergo subtype-specific transcriptional changes in Alzheimer's disease. Neuron 110:1788-1805.e10. https://doi.org/10.1016/j.neuron.2022.03.008

2. Hafemeister C, Satija R (2019) Normalization and variance stabilization of single-cell RNA-seq data using regularized negative binomial regression. Genome Biol 20:296. https://doi.org/10.1186/s13059-019-1874-1

3. Stuart T, Butler A, Hoffman P, Hafemeister C, Papalexi E, Mauck WM, Hao Y, Stoeckius M, Smibert P, Satija R (2019) Comprehensive Integration of Single-Cell Data. Cell 177:1888-1902.e21. https://doi.org/10.1016/j.cell.2019.05.031

4. Morabito S, Miyoshi E, Michael N, Shahin S, Martini AC, Head E, Silva J, Leavy K, Perez-Rosendahl M, Swarup V (2021) Single-nucleus chromatin accessibility and transcriptomic characterization of Alzheimer's disease. Nat Genet 53:1143–1155. https://doi.org/10.1038/s41588-021-00894-z
